# Supplementary material for: Plant organ evolution revealed by phylotranscriptomics in Arabidopsis thaliana
Source: Sci Rep. 2017 Aug 8;7:7567. doi: 10.1038/s41598-017-07866-6 (PMC5548721; doi:10.1038/s41598-017-07866-6)
Supplement: Supplementary file 1 — Supplementary Information [file 41598_2017_7866_MOESM1_ESM.pdf]

Supplemental figures and tables

Plant organ evolution revealed by phylotranscriptomics in *Arabidopsis thaliana*

Li Lei[1]*, Joshua G Steffen[2], Edward J Osborne[3], Christopher Toomajian[1]*
[1] Kansas State University, Department of Plant Pathology, Manhattan, KS, 66506
[2] Colby-Sawyer College, Natural Sciences Department, New London, NH, 03257
[3] University of Utah, Department of Biology, Salt Lake City, UT, 84111
*To whom correspondence should be addressed: toomajia@ksu.edu & llei@umn.edu

Figure S1 The distribution of dN/dS ratio based on genome comparisons of *A. thaliana* with *T. halophila* (A), *C. rubella* (B), and *B. rapa* (C).
Figure S2 The transcriptome age index (TDI) profile of three organs across 19 accessions based on three different genome comparisons: *A. thaliana* and *T. halophila* (A & B), *A. thaliana* and *C. rubella* (C & D), *A. thaliana* and *B. rapa* (E & F). Left column is sense (A, C & E), while right panel is antisense (B, D & F).
Figure S3 Mean relative sense (left column) and antisense (right column) expression levels of evolutionarily old (PS1-PS3) and young (PS4-PS12) genes in different organs in 19 accessions. The comparisons of relative antisense expression levels between old and young genes in different organs were performed by t test: *** means p-value <0.001; ** means p-value < 0.01; * means p-value <0.05; no * means not significant.
Figure S4. Phylostratigraphic analysis of antisense gene expression in *A. thaliana* seedling, root and flower. RSF: genes with expression shared by Root, Seedling and Flower; S: Seedling-specific expressed genes; R: Root-specific expressed genes; F: Flower-specific expressed genes; Total: all the protein coding genes with an assigned phylostratum. Gray line: A log-odds of zero, which corresponds to the actual number of genes in each phylostratum equaling the predicted number. (* P <0.05, ** P <0.01 and *** P <0.001).
Table S1 The enrichment of antisense expression in organ-specific expressed genes. se: sense; as: antisense; se_prop: the proportion of genes with sense expression; as_actual: the number of genes with antisense expression; RSF: genes with expression shared by Root, Seedling and Flower; S: Seedling-specific expressed genes; R: Root-specific expressed genes; F: Flower-specific expressed genes; RS: genes with expression shared by Root and Seedling; FS: genes with expression shared by Flower and Seedling; FR: genes with expression shared by Flower and Root.
Table S2 Average TAI and standard error of 19 accessions done by bootstrap 1000 times. se: sense; as: antisense.
Table S3 P-value obtained from Mann-Whitney test for TAI for 19 accessions. se: sense; as: antisense.
Table S4 Average TDI (*A. thaliana* vs. *A. lyrata*) and standard error of 19 accessions done by bootstrap 1000 times. se: sense; as: antisense.
Table S5 P-value obtained from Mann-Whitney test for TDI (*A. thaliana* vs. *A. lyrata*) for 19 accessions. se: sense; as: antisense.
Table S6 Average TDI (*A. thaliana* vs. *T. halophila*) and standard error of 19 accessions done by bootstrap 1000 times. se: sense; as: antisense.

Table S7 P-value obtained from Mann-Whitney test for TDI (*A. thaliana* vs. *T. halophila*) for 19 accessions. se: sense; as: antisense.
Table S8 Average TDI (*A. thaliana* vs. *C. rubella*) and standard error of 19 accessions done by bootstrap 1000 times. se: sense; as: antisense.
Table S9 P-value obtained from Mann-Whitney test for TDI (*A. thaliana* vs. *C. rubella*) for 19 accessions
Table S10 Average TDI (*A. thaliana* vs. *B. rapa*) and standard error of 19 accessions done by bootstrap 1000 times. se: sense; as: antisense.
Table S11 P-value obtained from Mann-Whitney test for TDI (*A. thaliana* vs. *B. rapa*) for 19 accessions
Table S12 GO terms of Floral bud-specific sense expressed genes
Table S13 GO terms of Root-specific sense expressed genes
Table S14 GO terms for genes sense expressed in all three organs
Table S15 GO terms for genes in PS3 (Viridiplantae)
Table S16 GO terms for genes in PS4 (Embryophyta)
Table S17 GO terms for genes in PS5 (Tracheophyta)
Table S18 GO terms for genes in PS6 (Magnoliophyta)
Table S19 GO terms for genes in PS9 (Rosids)
Table S20 GO terms for genes in PS10 (Brassicales)
Table S21 GO terms for genes in PS11 (Arabidopsis)
Table S22 GO terms for genes in PS12 (Arabidopsis thaliana)
Table S23 GO terms for genes with dN/dS ratio >1 (compared with *Arabidopsis lyrata*)
Table S24 The phylostrata of genes with dN/dS>1 compared with *A. lyrata*

Supplementary dataset1 Normalized expression (sense and antisense) data from root, seedling and floral bud for 19 accessions (https://github.com/lilei1/TAI_TDI_A.thaliana).

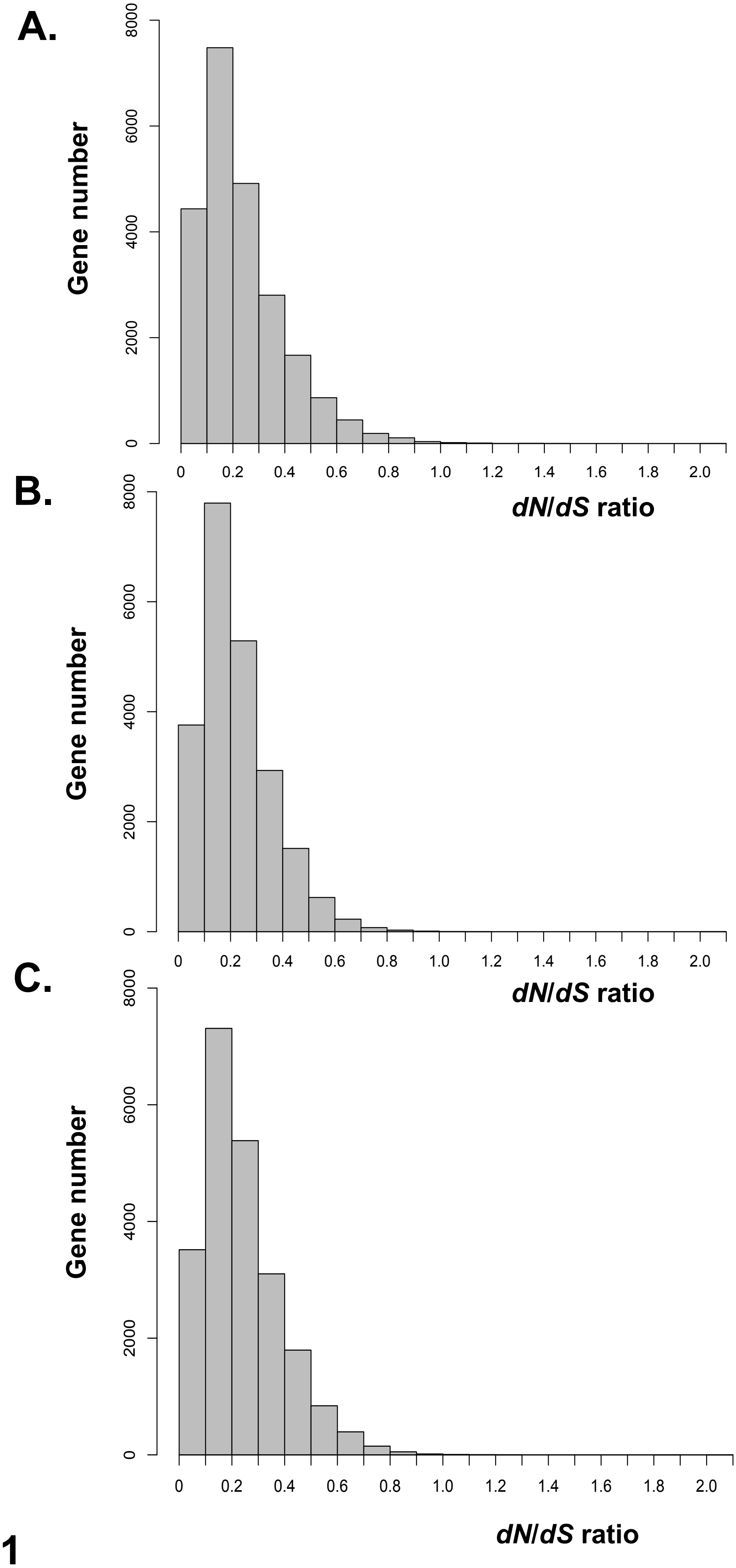
A.
B.
C.
Gene number
0
2000
4000
6000
8000
0
0.2
0.4
0.6
0.8
1.0
1.2
1.4
1.6
1.8
2.0
dN/dS ratio

Figure S1

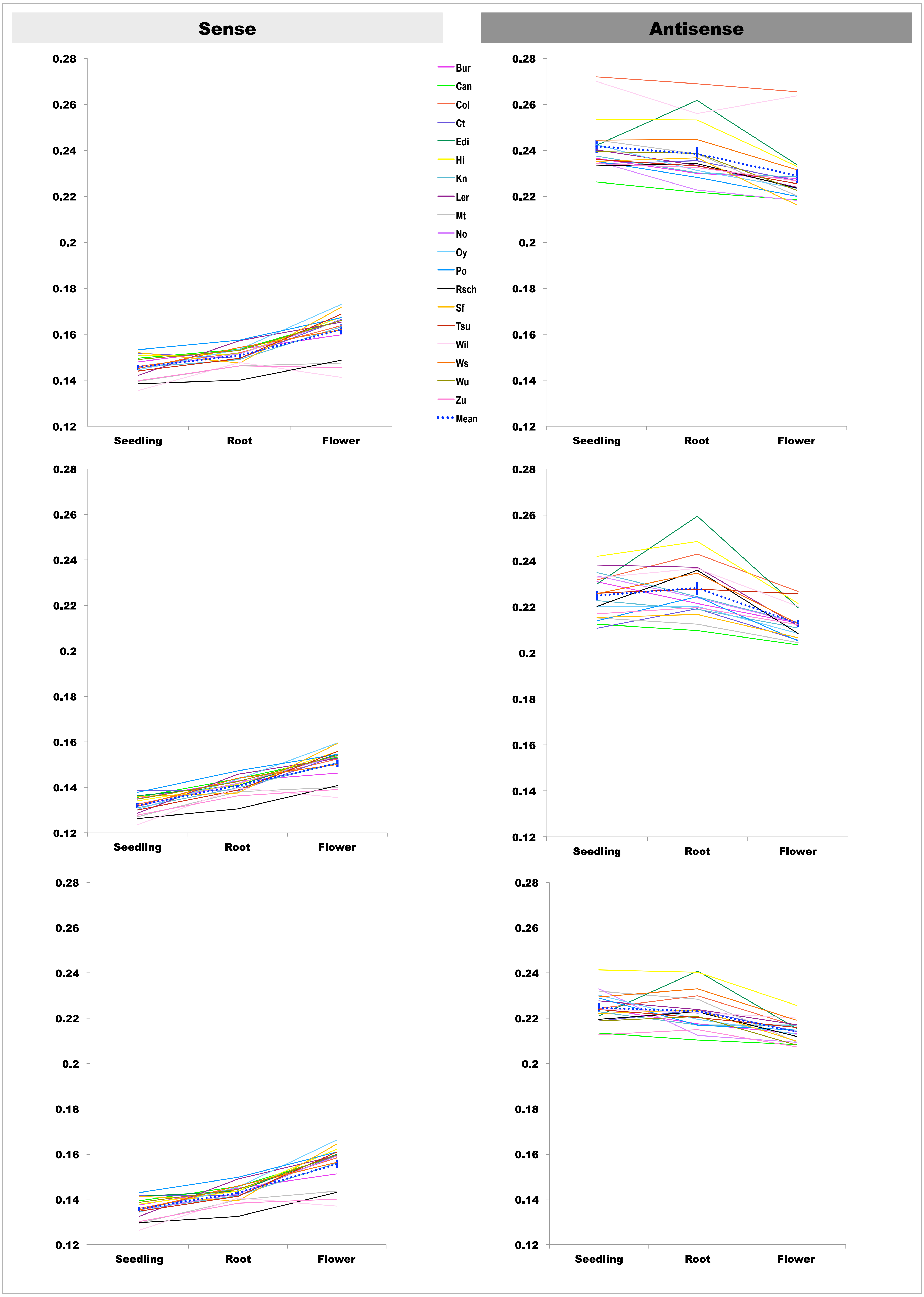
Sense
Antisense
Bur
Can
Col
Ct
Edi
Hi
Kn
Ler
Mt
No
Oy
Po
Rsch
Sf
Tsu
Wil
Ws
Wu
Zu
Mean
0.28
0.26
0.24
0.22
0.2
0.18
0.16
0.14
0.12
Seedling
Root
Flower

Figure S2

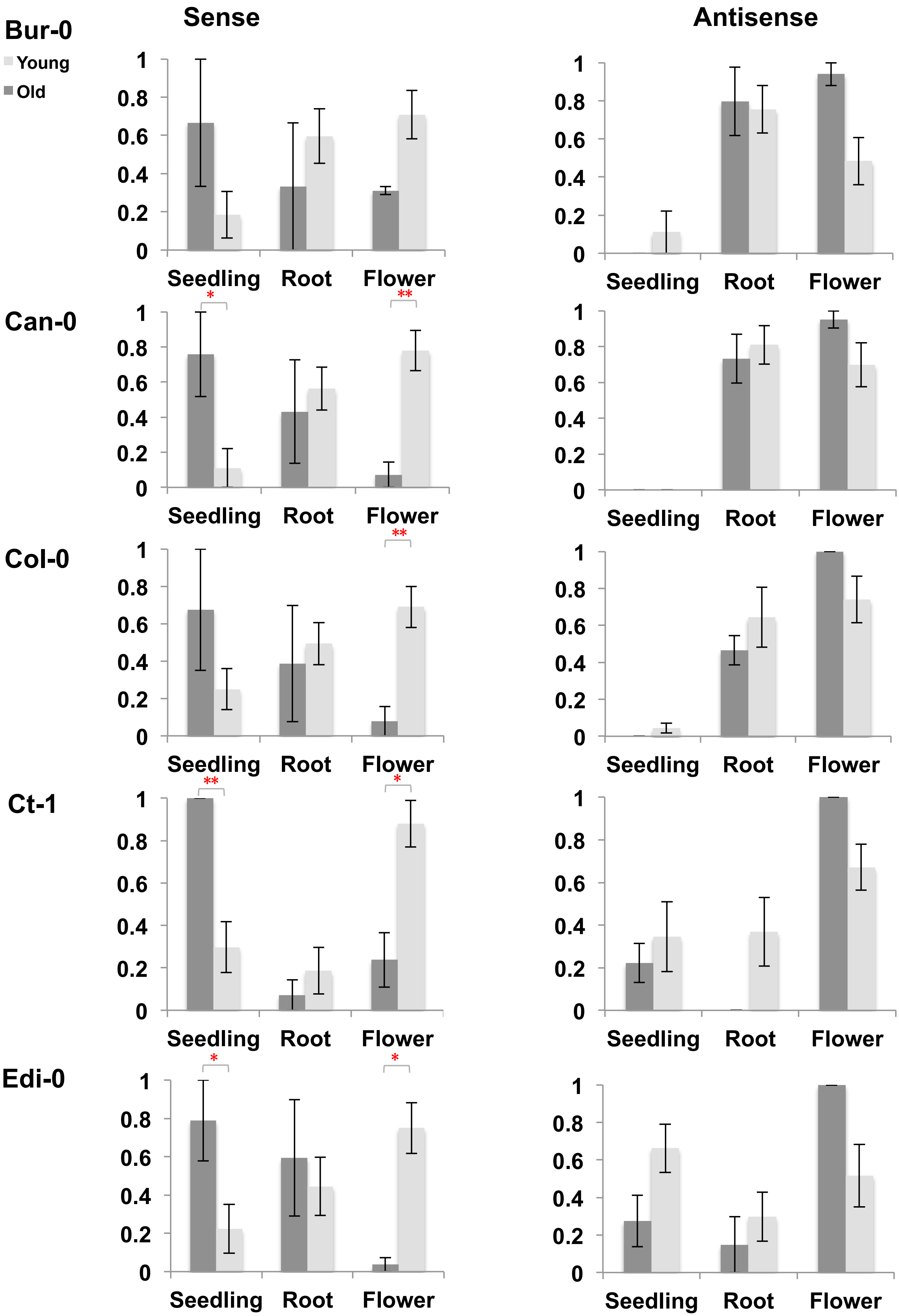

Sense
Antisense
Bur-0
Young
Old
Can-0
Col-0
Ct-1
Edi-0
1
0.8
0.6
0.4
0.2
0
Seedling
Root
Flower
*
**

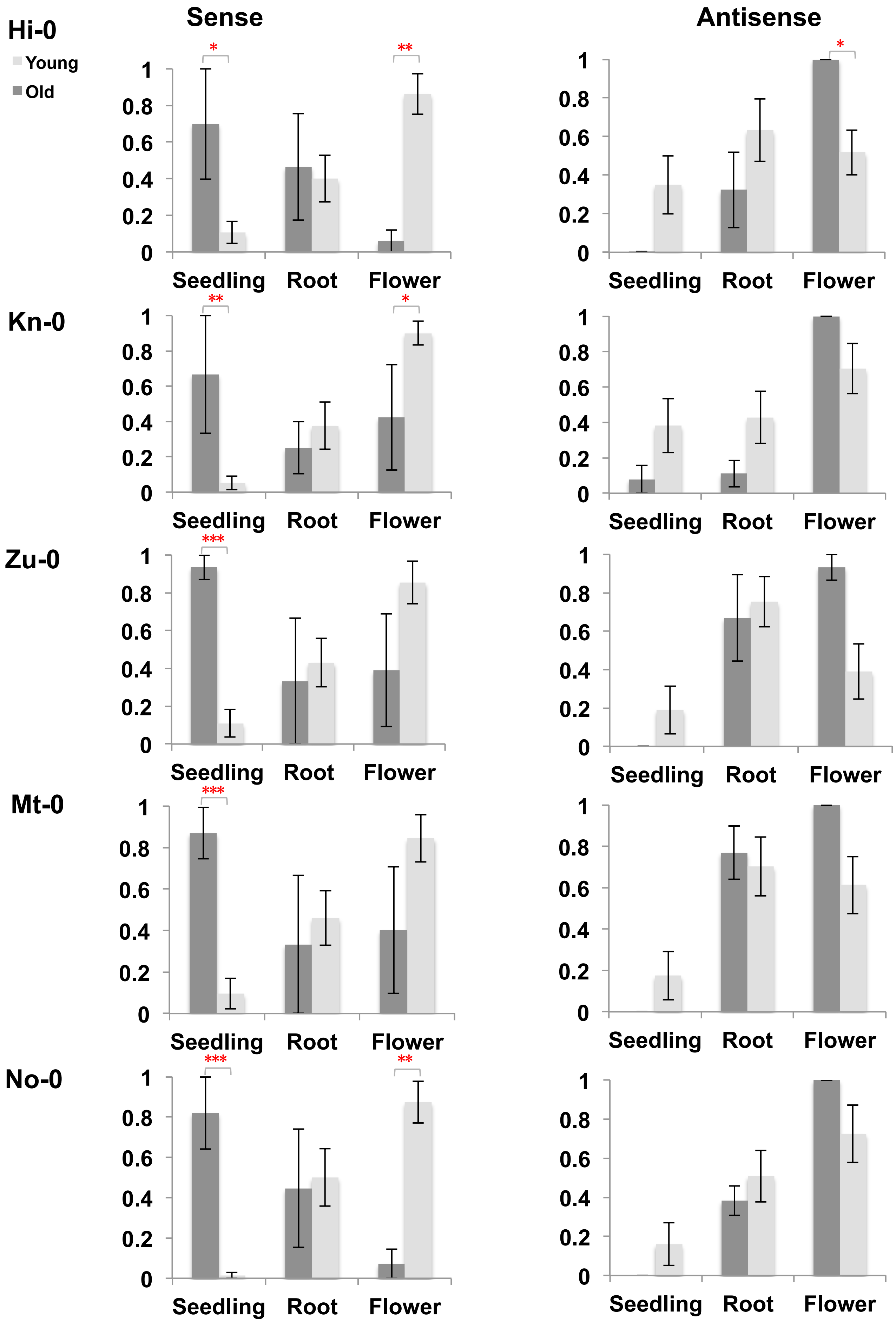

Sense
Antisense
Hi-0
Young
Old
Kn-0
Zu-0
Mt-0
No-0
Seedling
Root
Flower

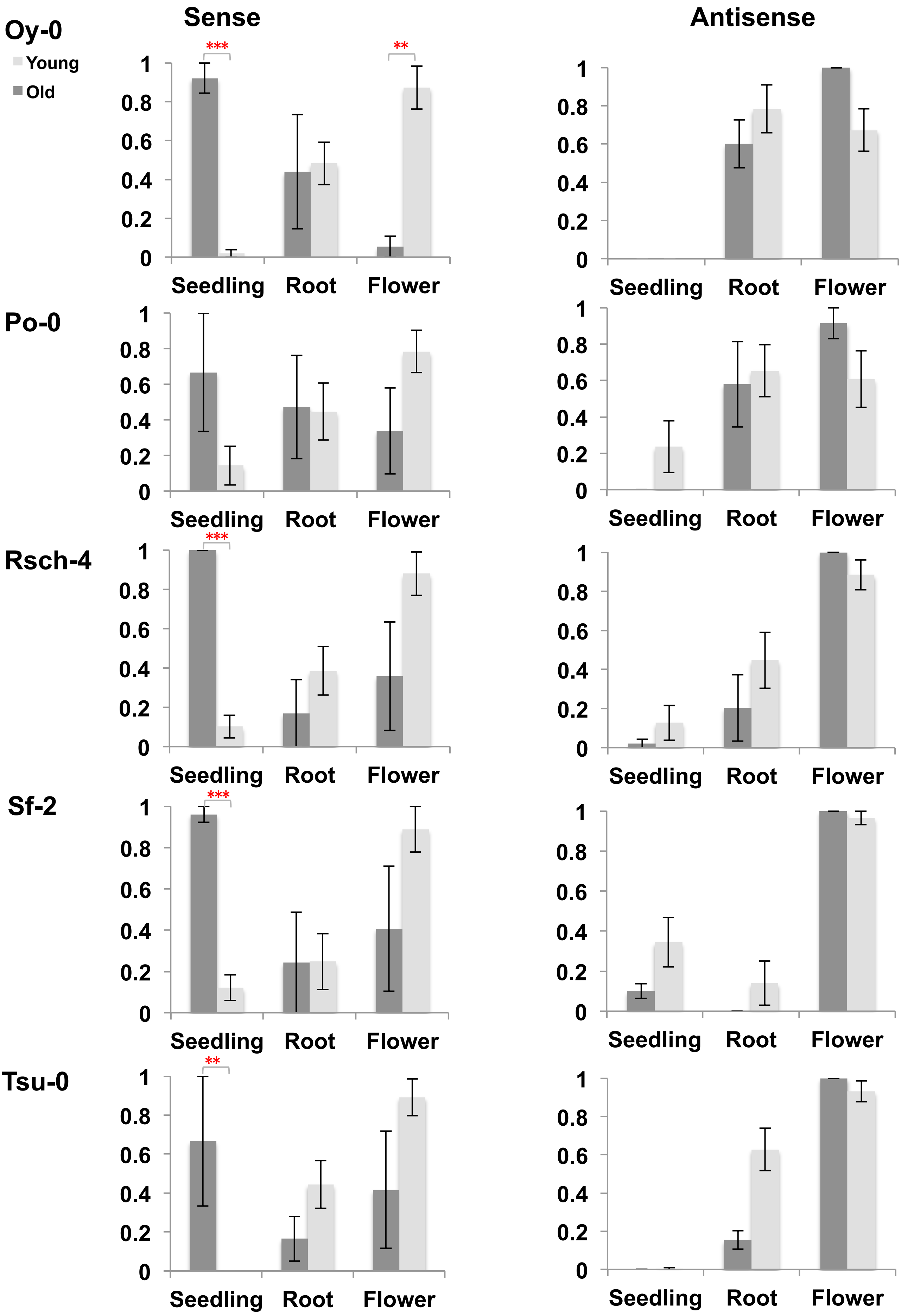
Sense
Antisense
Oy-0
Young
Old
Po-0
Rsch-4
Sf-2
Tsu-0
***
**
0
0.2
0.4
0.6
0.8
1
Seedling
Root
Flower

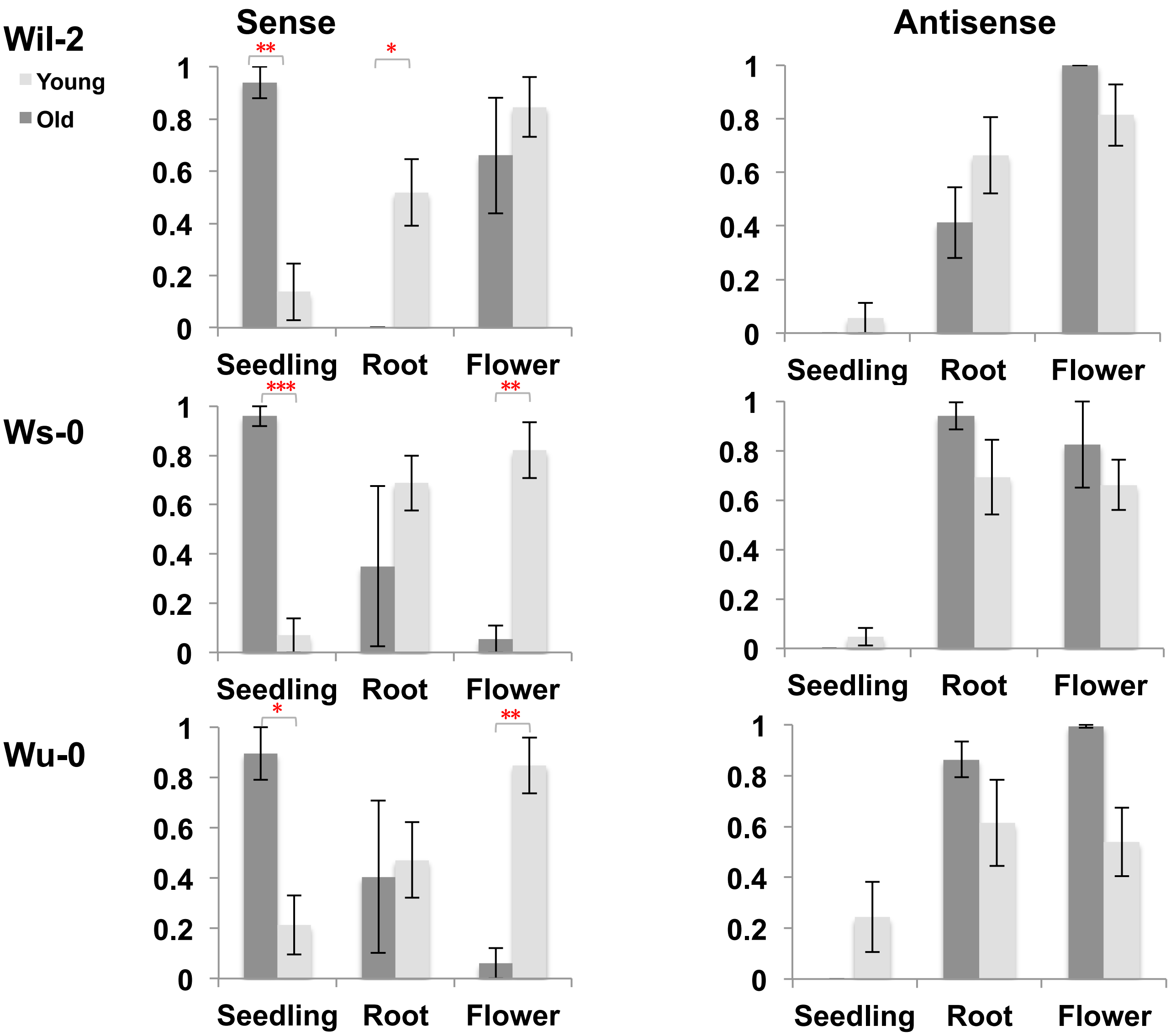

Sense
Antisense
Wil-2
Young
Old
Ws-0
Wu-0
1
0.8
0.6
0.4
0.2
0
Seedling
Root
Flower
**
*
***
**
*
**

Figure S3

9103	6503	782	2635	846	1571	282	276	352	970	188	1752	Total
716	438	63	275	95	116	29	22	18	48	6	156	RSF
519	346	57	132	42	61	6	12	15	26	2	81	F
394	230	50	149	35	44	12	18	14	40	2	59	R
145	110	17	43	16	16	3	3	4	11	1	23	S

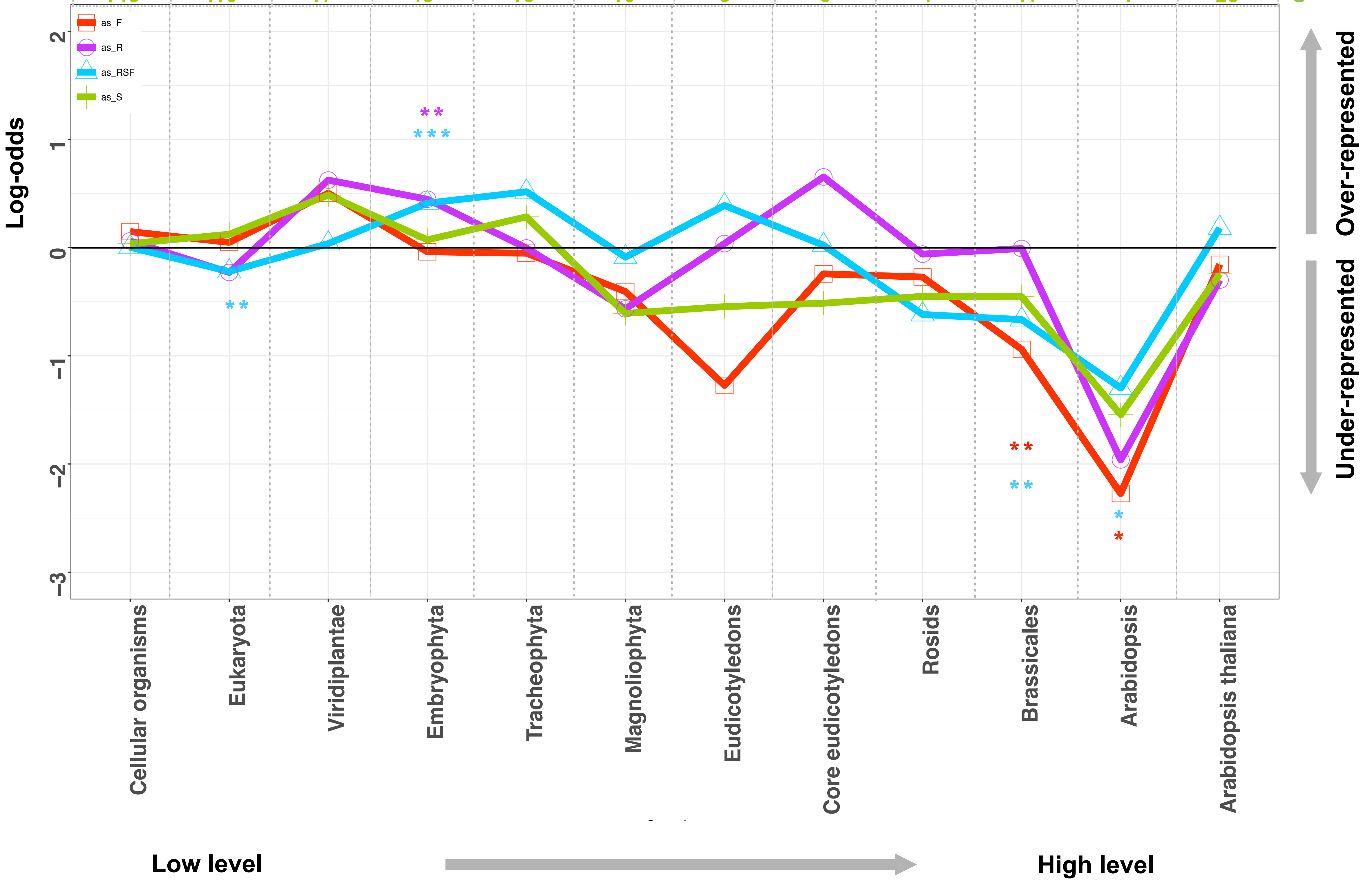

Figure S4

Table S1 Then enrichment of antisense expression in organ specific expressed genes

Categories	#Sense	Se_prop	#Antisense	#Expected_as	Enrichment	P-value
R	1488	0.06759028	1111	521.9321372	2.12862922	***
S	191	0.0086759	434	66.99532137	6.47806431	***
F	1486	0.06749943	1418	521.2306155	2.72048486	***
RS	335	0.0152169	192	117.504883	1.63397465	***
FS	916	0.04160799	747	321.2969339	2.32495216	***
FR	694	0.03152396	616	243.4280263	2.5305221	***
RSF	16905	0.76788553	2204	5929.612083	0.37169379	***
Sum	22015		6722			

#Sense: The number of genes with sense expression

Se_prop: The proportion of genes with sense expression

#Antisense: The number of genes with antisense expression

#Expected_as: The expeted number of genes with antisense expression

Enrichment: The enrichment of genes with antisense expression to expected

Table S2 Average TAI and standard error _

Strains	Seedling_se	Root_se	Flower_se	Seedling_as	Root_as	Flower_as
**Bur-0**	**2.235872±0.001824599**	**2.51009±0.002028479**	**2.419093±0.001542888**	4.151377±0.009239946	4.236548±0.007943619	4.579405±0.01294164
Can-0	2.288676±0.001778029	2.561527±0.0022962	2.71685±0.002007312	4.284865±0.0129891	4.236548±0.009717936	4.579405±0.01659421
Col-0	2.228196±0.00147881	2.550572±0.002092515	2.734957±0.002049928	**6.122459±0.04131502**	**5.738568±0.03528634**	**6.28172±0.0423672**
Ct-1	2.346395±0.002620549	2.457332±0.002036376	2.721089±0.002046879	4.070135±0.0086997	4.215621±0.007567985	4.164578±0.007975459
Edi-0	2.352741±0.001853317	2.515859±0.002240583	2.698026±0.001771826	4.359253±0.009947839	4.119009±0.007729609	4.819295±0.01672777
Hi-0	2.227727±0.001615279	2.497287±0.001988035	2.790117±0.002255361	4.485354±0.01105861	4.305913±0.008304569	4.427964±0.01157827
Kn-0	2.212304±0.001730698	2.507491±0.002352795	2.72712±0.002170534	4.090209±0.00567677	4.000225±0.00433171	4.038353±0.00503836
Ler-0	2.186578±0.001764366	2.633172±0.002901368	2.792041±0.002146274	4.979422±0.01928831	4.688942±0.01300373	5.024142±0.01789582
Mt-0	2.186935±0.002624901	2.474023±0.002836212	2.497827±0.006289435	4.234841±0.01231415	4.182637±0.01171844	4.269846±0.01903384
No-0	2.177128±0.001472978	2.527872±0.002113878	2.77769±0.002142789	4.63568±0.01601086	4.263322±0.01149144	4.569337±0.01684334
Oy-0	2.208795±0.001735602	2.597553±0.002453847	2.915568±0.002452898	3.89819±0.005346485	3.994058±0.005697879	4.560598±0.02129871
Po-0	2.37393±0.002175111	2.593234±0.002265206	2.730598±0.002165136	4.244764±0.01015635	4.126906±0.006825637	4.786748±0.01871558
Rsch-4	2.159457±0.002128699	2.427578±0.002662789	2.622048±0.006763947	4.244764±0.01472517	4.772266±0.01847897	4.460555±0.01559564
Sf-2	2.337635±0.002039641	2.462658±0.001942459	2.978038±0.002842237	4.369816±0.007957225	4.447095±0.01025887	4.175661±0.008419504
Tsu-0	2.182904±0.001762086	2.576274±0.002832644	2.983051±0.003178438	4.566031±0.01380469	4.528638±0.01177054	5.144075±0.020372
**Wil-2**	**2.10793±0.002396696**	**2.505253±0.00299629**	**2.436049±0.006737725**	**5.75043±0.04388633**	**4.992194±0.02767422**	**5.527462±0.03944152**
Ws-0	2.210802±0.001585576	2.595913±0.002258261	2.634982±0.001987447	4.319492±0.01166669	4.302528±0.009071082	4.574392±0.0162471
Wu-0	2.26343±0.001654281	2.541914±0.002175789	2.701005±0.001914301	4.221004±0.01104913	4.121059±0.008924967	4.960231±0.02131581
**Zu-0**	**2.153586±0.002809704**	**2.476766±0.002684091**	**2.470865±0.006678977**	4.178452±0.01049238	4.232525±0.007977896	4.201074±0.01176444

Table S3 P-value obtained from Mann-Whitney test for TAI for 19 accessions

Strains	Seedling_se vs. Flower_se	Seedling_se vs. Root_se	Root_se vs. Flower_se	Seedling_as vs. Flower_as	Seedling_as vs. Root_as	Root_as vs. Flower_as
Bur-0	< 2.2e-16	< 2.2e-16	< 2.2e-16	< 2.2e-16	< 2.2e-16	1.33E-12
Can-0	< 2.2e-16	< 2.2e-16	< 2.2e-16	< 2.2e-16	0.3835	< 2.2e-16
Col-0	< 2.2e-16	< 2.2e-16	< 2.2e-16	0.009251	4.365E-15	< 2.2e-16
Ct-1	< 2.2e-16	< 2.2e-16	< 2.2e-16	1.37E-15	< 2.2e-16	9.27E-07
Edi-0	< 2.2e-16	< 2.2e-16	< 2.2e-16	< 2.2e-16	< 2.2e-16	< 2.2e-16
Hi-0	< 2.2e-16	< 2.2e-16	< 2.2e-16	0.000009504	< 2.2e-16	9.28E-16
Kn-0	< 2.2e-16	< 2.2e-16	< 2.2e-16	6.671E-11	< 2.2e-16	4.35E-08
Ler-0	< 2.2e-16	< 2.2e-16	< 2.2e-16	0.0001152	< 2.2e-16	< 2.2e-16
Mt-0	< 2.2e-16	< 2.2e-16	0.0000597	0.3003	0.000368	0.09652
No-0	< 2.2e-16	< 2.2e-16	< 2.2e-16	0.003112	< 2.2e-16	< 2.2e-16
Oy-0	< 2.2e-16	< 2.2e-16	< 2.2e-16	< 2.2e-16	< 2.2e-16	< 2.2e-16
Po-0	< 2.2e-16	< 2.2e-16	< 2.2e-16	< 2.2e-16	< 2.2e-16	< 2.2e-16
Rsch-4	< 2.2e-16	< 2.2e-16	< 2.2e-16	0.005691	< 2.2e-16	< 2.2e-16
Sf-2	< 2.2e-16	< 2.2e-16	< 2.2e-16	< 2.2e-16	1.186E-11	< 2.2e-16
Tsu-0	< 2.2e-16	< 2.2e-16	< 2.2e-16	< 2.2e-16	0.02713	< 2.2e-16
Wil-2	< 2.2e-16	< 2.2e-16	4.028E-12	0.001672	< 2.2e-16	< 2.2e-16
Ws-0	< 2.2e-16	< 2.2e-16	< 2.2e-16	< 2.2e-16	0.3976	< 2.2e-16
Wu-0	< 2.2e-16	< 2.2e-16	< 2.2e-16	< 2.2e-16	1.446E-10	< 2.2e-16
Zu-0	< 2.2e-16	< 2.2e-16	0.9695	0.4155	0.000005651	0.001851

se: sense

as:antisense

Bold: the accessions with the order of TAI not follow Seedling<Root <Flower

Table S4 Average TDI (*A. thaliana* vs. *A. lyrata*) and standard error of 19 accessions done by bootstrap 1000 times

Strains	Seedling_se	Root_se	Flower_se	Seedling_as	Root_as	Flower_as
Bur-0	0.14245±0.00009967509	0.1507515±0.0001328056	0.1551607±0.00009920188	0.2397432±0.000305531	0.2362117±0.0002532825	0.2291589±0.0002065077
Can-0	0.1448816±0.0001028612	0.1504544±0.000109791	0.1612138±0.00008721394	0.2266502±0.0001971957	0.2323304±0.0002093004	0.2211302±0.000158287
Col-0	0.1405456±0.0001081164	0.1484947±0.0001037258	0.160652±0.00009545081	**0.313807±0.001764786**	**0.3037431±0.001481361**	**0.3098583±0.001912762**
**Ct-1**	**0.1466439±0.0001050695**	**0.1451369±0.00009460885**	**0.1626047±0.00009138141**	0.2304861±0.0002594213	0.2418359±0.0002922339	0.2262906±0.0002039289
Edi-0	0.1433338±0.0001171703	0.1479693±0.0001095287	0.1629489±0.00008643823	0.2407541±0.0002880869	0.2528557±0.0006442686	0.2274712±0.0002192744
Hi-0	0.1414045±0.0001035537	0.1482953±0.0001001187	0.1651423±0.00009705413	0.2543489±0.0006039117	0.25131±0.0006229443	0.2351171±0.0003651184
Kn-0	0.1378525±0.0001108046	0.1466899±0.00009474784	0.1595936±0.00009332264	0.2362448±0.0002239791	0.2263663±0.0001935801	0.2225708±0.0001651948
Ler-0	0.1361127±0.0001071449	0.153523±0.0001566708	0.1618278±0.00009421769	0.239005±0.0003604362	0.2316258±0.0002776347	0.2168242±0.0001652712
**Mt-0**	**0.1335666±0.0002312816**	**0.1431191±0.0002026566**	**0.1413431±0.0005387666**	0.242145±0.0005022001	0.2405211±0.0004277335	0.222844±0.0002300149
No-0	0.1394229±0.0001071201	0.1497731±0.0001017122	0.1619712±0.00008303046	0.2335689±0.0002230178	0.225146±0.0001743687	0.2222003±0.0002490992
Oy-0	0.1383133±0.0001030548	0.1513057±0.00009778876	0.1686587±0.0001034443	0.2383208±0.0002624191	0.2284029±0.0002448673	0.2273007±0.0001828152
Po-0	0.1451254±0.0001165205	0.1539065±0.0001219918	0.1640251±0.00009881445	0.2397556±0.0002671046	0.2335208±0.000210771	0.2225467±0.0001510594
Rsch-4	0.1323313±0.0001816495	0.1350558±0.0001618419	0.1421393±0.0005568043	0.2290515±0.0001761514	0.2331093±0.0002003178	0.2163604±0.0001632581
Sf-2	0.1428623±0.0001234334	0.1445196±0.00009028205	0.1687531±0.0001106224	0.2433385±0.0003438297	0.2501527±0.0003467926	0.2258401±0.0002439503
Tsu-0	0.1379193±0.00009777936	0.1454028±0.000105443	0.1664056±0.0001126668	0.2353666±0.0002984395	0.2354193±0.0002745418	0.2240801±0.000207546
**Wil-2**	**0.1292446±0.0002018473**	**0.1448624±0.0001834546**	**0.1373399±0.0005957278**	**0.3085742±0.001708795**	**0.2778912±0.001108354**	**0.2956641±0.001567156**
Ws-0	0.1395373±0.0001057092	0.1516594±0.0001086458	0.1596448±0.0001005761	0.2383221±0.0003391967	0.2394016±0.0004227876	0.2268806±0.0002222839
Wu-0	0.1424717±0.0001009597	0.1498538±0.0001182135	0.1617062±0.00009299547	0.2420583±0.0002905034	0.2409571±0.0003499247	0.2307049±0.000289264
**Zu-0**	**0.1329033±0.000262557**	**0.1416976±0.0001700377**	**0.1403276±0.0005684442**	0.2282296±0.000184482	0.2292165±0.0001826258	0.2240903±0.0001609507

Table S5 P-value obtained from Mann-Whitney test for TDI for 19 accessions

Strains	Seedling_se vs. Flower_se	Seedling_se vs. Root_se	Root_se vs. Flower_se	Seedling_as vs. Flower_as	Seedling_as vs. Root_as	Root_as vs. Flower_as
Bur-0	< 2.2e-16	< 2.2e-16	< 2.2e-16	< 2.2e-16	< 2.2e-16	< 2.2e-16
Can-0	< 2.2e-16	< 2.2e-16	< 2.2e-16	< 2.2e-16	< 2.2e-16	< 2.2e-16
Col-0	< 2.2e-16	< 2.2e-16	< 2.2e-16	0.02095	4.421E-08	0.001336
Ct-1	< 2.2e-16	< 2.2e-16	< 2.2e-16	< 2.2e-16	< 2.2e-16	< 2.2e-16
Edi-0	< 2.2e-16	< 2.2e-16	< 2.2e-16	< 2.2e-16	< 2.2e-16	< 2.2e-16
Hi-0	< 2.2e-16	< 2.2e-16	< 2.2e-16	< 2.2e-16	0.000196	< 2.2e-16
Kn-0	< 2.2e-16	< 2.2e-16	< 2.2e-16	< 2.2e-16	< 2.2e-16	< 2.2e-16
Ler-0	< 2.2e-16	< 2.2e-16	< 2.2e-16	< 2.2e-16	< 2.2e-16	< 2.2e-16
Mt-0	< 2.2e-16	< 2.2e-16	0.03208	< 2.2e-16	0.1039	< 2.2e-16
No-0	< 2.2e-16	< 2.2e-16	< 2.2e-16	< 2.2e-16	< 2.2e-16	< 2.2e-16
Oy-0	< 2.2e-16	< 2.2e-16	< 2.2e-16	< 2.2e-16	< 2.2e-16	0.003447
Po-0	< 2.2e-16	< 2.2e-16	< 2.2e-16	< 2.2e-16	< 2.2e-16	< 2.2e-16
Rsch-4	< 2.2e-16	< 2.2e-16	< 2.2e-16	< 2.2e-16	< 2.2e-16	< 2.2e-16
Sf-2	< 2.2e-16	< 2.2e-16	< 2.2e-16	< 2.2e-16	< 2.2e-16	< 2.2e-16
Tsu-0	< 2.2e-16	< 2.2e-16	< 2.2e-16	< 2.2e-16	0.8272	< 2.2e-16
Wil-2	< 2.2e-16	< 2.2e-16	< 2.2e-16	< 2.2e-16	< 2.2e-16	< 2.2e-16
Ws-0	< 2.2e-16	< 2.2e-16	< 2.2e-16	< 2.2e-16	0.3161	< 2.2e-16
Wu-0	< 2.2e-16	< 2.2e-16	< 2.2e-16	< 2.2e-16	0.001283	< 2.2e-16
Zu-0	< 2.2e-16	< 2.2e-16	0.001942	< 2.2e-16	0.0004033	< 2.2e-16

se: sense

as:antisense

Bold: the accessions with the order of TDI not follow Seedling<Root <Flower

Table S6 Average TDI (*A. thaliana* vs. *T. halophila*) and standard error of 19 accessions done by bootstrap 1000 times. se: sense; as: antisense.

Strains	Seedling_se	Root_se	Flower_se	Seedling_as	Root_as	Flower_as
Bur-0	0.1479236±0.00008737239	0.1535394±0.00009258473	0.159769±0.00008851699	0.2365805±0.0003076433	0.2300178±0.0003139547	0.2277985±0.0002079957
Can-0	0.1495941±0.00008265894	0.1534603±0.00007842097	0.1659848±0.00008105855	0.2262042±0.0001920458	0.2218326±0.0002158378	0.2184034±0.0001751258
Col-0	0.1459145±0.00009109964	0.1519746±0.00007231448	0.1637226±0.00008361986	0.271952±0.0007570257	0.269062±0.0007106308	0.2653682±0.0008448697
**Ct-1**	**0.1517936±0.00008644335**	**0.1495132±0.00007382281**	**0.1664243±0.00008096476**	0.2341732±0.0002164539	0.2355908±0.0001977545	0.2268008±0.0001798704
Edi-0	0.1506069±0.0001082295	0.1514813±0.00007714838	0.1674209±0.0000806409	0.2423056±0.0002141285	0.261798±0.000724446	0.2338165±0.0002306705
Hi-0	0.1481339±0.00008775198	0.1512291±0.0000734821	0.1685499±0.00007685105	0.253455±0.0006797984	0.2531262±0.0007153367	0.2330623±0.0003791502
Kn-0	0.1455066±0.0000901326	0.1490569±0.00007506032	0.1633116±0.00007195773	0.2375083±0.0001692511	0.2302342±0.0001671396	0.2283596±0.0001514815
Ler-0	0.1421528±0.0000950824	0.1573965±0.0001095575	0.1652564±0.00008420981	0.2401756±0.0003748605	0.2334564±0.0002155192	0.2239606±0.0001662752
Mt-0	0.1395104±0.000197143	0.1461804±0.0001579014	0.1474809±0.0004762177	0.2445492±0.0006131327	0.2385891±0.0005649536	0.2202798±0.0002974435
No-0	0.1453099±0.00009292818	0.1514218±0.00007636001	0.1663168±0.00007486371	0.2352069±0.0001992687	0.2226977±0.000204009	0.2181254±0.0002009315
Oy-0	0.1445563±0.00008842812	0.1535355±0.00007718884	0.1730297±0.00009149511	0.2424234±0.0002594734	0.2313378±0.0003094688	0.2234636±0.0002205839
Po-0	0.1533098±0.00009931394	0.1574782±0.00008498234	0.1671584±0.00007531315	0.2351226±0.0001993973	0.2283359±0.0002337535	0.2200243±0.0001679641
Rsch-4	0.138548±0.0001536478	0.1399398±0.0001394105	0.1486678±0.0004915363	0.2333352±0.0001627737	0.2341815±0.0001545698	0.2235863±0.0001907249
**Sf-2**	**0.1520513±0.0001162356**	**0.1474865±0.00006646744**	**0.1717227±0.0000998949**	0.2351619±0.000262302	0.2367207±0.0003642021	0.2162806±0.0002467111
Tsu-0	0.1439233±0.00009028126	0.1496379±0.00007960853	0.1688926±0.00008895926	0.2360985±0.0001783884	0.2331556±0.0001754618	0.2255065±0.0001748173
**Wil-2**	**0.135418±0.0001732926**	**0.1478733±0.0001484483**	**0.1411988±0.0005112911**	0.2699616±0.0007524913	0.2559973±0.0005399869	0.2637538±0.0007038867
Ws-0	0.1451508±0.00009273093	0.1543786±0.00007943041	0.1621296±0.00008086476	0.2445067±0.0003803966	0.2446465±0.0005122208	0.2315646±0.0002443083
Wu-0	0.1489985±0.00008741756	0.1531205±0.00008538122	0.1660425±0.00008329231	0.2394713±0.0002784902	0.2388367±0.0004271273	0.2226087±0.0002830537
**Zu-0**	**0.1398365±0.0002288199**	**0.1462918±0.0001379558**	**0.1456318±0.0005134242**	0.2343336±0.000175763	0.2325294±0.0001617519	0.2266185±0.0001606168

Table S7 P-value obtained from Mann-Whitney test for TDI for 19 accessions

Strains	Seedling_se vs. Flower_se	Seedling_se vs. Root_se	Root_se vs. Flower_se	Seedling_as vs. Flower_as	Seedling_as vs. Root_as	Root_as vs. Flower_as
Bur	< 2.2e-16	< 2.2e-16	< 2.2e-16	< 2.2e-16	< 2.2e-16	2.023E-07
Can	< 2.2e-16	< 2.2e-16	< 2.2e-16	< 2.2e-16	< 2.2e-16	< 2.2e-16
Col	< 2.2e-16	< 2.2e-16	< 2.2e-16	1.364E-09	0.0008727	0.01706
Ct	< 2.2e-16	< 2.2e-16	< 2.2e-16	< 2.2e-16	0.00002041	< 2.2e-16
Edi	< 2.2e-16	1.459E-09	< 2.2e-16	< 2.2e-16	< 2.2e-16	< 2.2e-16
Hi	< 2.2e-16	< 2.2e-16	< 2.2e-16	< 2.2e-16	0.4767	< 2.2e-16
Kn	< 2.2e-16	< 2.2e-16	< 2.2e-16	< 2.2e-16	< 2.2e-16	5.721E-16
Ler	< 2.2e-16	< 2.2e-16	< 2.2e-16	< 2.2e-16	< 2.2e-16	< 2.2e-16
Mt	< 2.2e-16	< 2.2e-16	0.01788	< 2.2e-16	5.944E-11	< 2.2e-16
No	< 2.2e-16	< 2.2e-16	< 2.2e-16	< 2.2e-16	< 2.2e-16	< 2.2e-16
Oy	< 2.2e-16	< 2.2e-16	< 2.2e-16	< 2.2e-16	< 2.2e-16	< 2.2e-16
Po	< 2.2e-16	< 2.2e-16	< 2.2e-16	< 2.2e-16	< 2.2e-16	< 2.2e-16
Rsch	< 2.2e-16	9.575E-11	< 2.2e-16	< 2.2e-16	0.0005184	< 2.2e-16
Sf	< 2.2e-16	< 2.2e-16	< 2.2e-16	< 2.2e-16	0.0276	< 2.2e-16
Tsu	< 2.2e-16	< 2.2e-16	< 2.2e-16	< 2.2e-16	< 2.2e-16	< 2.2e-16
Wil	< 2.2e-16	< 2.2e-16	< 2.2e-16	1.183E-10	< 2.2e-16	< 2.2e-16
Ws	< 2.2e-16	< 2.2e-16	< 2.2e-16	< 2.2e-16	0.2904	< 2.2e-16
Wu	< 2.2e-16	< 2.2e-16	< 2.2e-16	< 2.2e-16	0.00557	< 2.2e-16
Zu	< 2.2e-16	< 2.2e-16	0.02684	< 2.2e-16	1.776E-12	< 2.2e-16

se: sense

as:antisense

Bold: the accessions with the order of TDI not follow Seedling<Root <Flower

Table S8 Average TDI (*A. thaliana* vs. *C. rubella*) and standard error of 19 accessions done by bootstrap 1000 times

Strains	Seedling_se	Root_se	Flower_se	Seedling_as	Root_as	Flower_as
Bur-0	0.1349752±0.0000902546	0.1427328±0.0000850831	0.1461944±0.00008845767	0.2309451±0.0004599588	0.2215211±0.0003730906	0.2128729±0.0002528567
Can-0	0.1356327±0.00007853659	0.1436779±0.00008192269	0.153375±0.00008071593	0.2124372±0.0001997973	0.209812±0.000201904	0.20347±0.0001517792
Col-0	0.1324246±0.00008198215	0.1417663±0.00007444586	0.1524974±0.00008806319	0.2316186±0.0003588102	0.2429358±0.0005308924	0.2266564±0.0003066678
**Ct-1**	**0.1384233±0.0000862507**	**0.1383936±0.00007687173**	**0.1541263±0.00007896567**	0.2107144±0.0002116679	0.2191769±0.0001949747	0.2055941±0.0001461933
Edi-0	0.1363529±0.00009226218	0.1408588±0.00008433486	0.1545017±0.00007601818	0.2300031±0.000337028	0.2594042±0.001010642	0.2197366±0.0003076051
Hi-0	0.1340138±0.0000802722	0.1413447±0.00007383421	0.155746±0.00007646857	0.2419579±0.0009455122	0.2484854±0.0009655924	0.2215404±0.0005006654
Kn-0	0.131037±0.00008446264	0.1396889±0.00007849373	0.1507711±0.00007492476	0.2227692±0.0002354823	0.2190282±0.0001961073	0.2108543±0.0001642648
Ler-0	0.1286191±0.000089756	0.1459063±0.0000998478	0.1527587±0.00008010895	0.2349065±0.000504574	0.2244192±0.0002664919	0.21314±0.0001898144
Mt-0	0.1270873±0.0001518001	0.1379919±0.0001392072	0.1399973±0.000383017	0.2381294±0.0008207294	0.2371145±0.0007155796	0.2117285±0.0003539722
No-0	0.1316303±0.0000866851	0.1416805±0.0000781719	0.1538664±0.0000750081	0.2152649±0.0002009655	0.2124014±0.000201947	0.2044566±0.0001909467
Oy-0	0.1303049±0.00008035961	0.1434124±0.0000772635	0.1595725±0.00008841373	0.2334961±0.000331466	0.2239938±0.0003532464	0.2130614±0.0002323188
Po-0	0.1377536±0.0000888787	0.1472939±0.0000882293	0.154442±0.00008416516	0.2201601±0.0002507734	0.2201565±0.0002582024	0.2084107±0.0001817187
Rsch-4	0.1262024±0.0001235878	0.1306361±0.0001160439	0.1407037±0.0003846126	0.2140211±0.0001823557	0.2245693±0.0002138006	0.2052545±0.0001465289
Sf-2	0.1359719±0.00009858807	0.1375355±0.00007450484	0.159357±0.00009733459	0.2202331±0.0003335166	0.2360682±0.000469302	0.2084781±0.0002791776
Tsu-0	0.1299953±0.00008505875	0.1387292±0.00007754555	0.1557074±0.00008420884	0.215467±0.0001636377	0.2166605±0.0001993921	0.2065983±0.0001510785
**Wil-2**	**0.1234725±0.0001333809**	**0.1397375±0.0001268305**	**0.1356558±0.0003969439**	0.2259262±0.0002768679	0.2276471±0.0003105307	0.2257127±0.0002463267
Ws-0	0.1317301±0.0000866889	0.1441533±0.00007831524	0.1499605±0.00007758801	0.232827±0.0005341945	0.2366124±0.0007000874	0.2201763±0.0003267906
Wu-0	0.1348346±0.00007944101	0.1426108±0.00008071579	0.1540402±0.0000849022	0.2258151±0.0003645034	0.234815±0.0005465712	0.2126198±0.0003389556
Zu-0	0.1277872±0.0001795896	0.1363834±0.0001221224	0.1389775±0.0004202181	0.2171101±0.0002111038	0.2196646±0.0002023862	0.2122449±0.00018681

Table S9 P-value obtained from Mann-Whitney test for TDI for 19 accessions

Strains	Seedling_se vs. Flower_se	Seedling_se vs. Root_se	Root_se vs. Flower_se	Seedling_as vs. Flower_as	Seedling_as vs. Root_as	Root_as vs. Flower_as
Bur-0	< 2.2e-16	< 2.2e-16	< 2.2e-16	< 2.2e-16	< 2.2e-16	< 2.2e-16
Can-0	< 2.2e-16	< 2.2e-16	< 2.2e-16	< 2.2e-16	< 2.2e-16	< 2.2e-16
Col-0	< 2.2e-16	< 2.2e-16	< 2.2e-16	< 2.2e-16	< 2.2e-16	< 2.2e-16
Ct-1	< 2.2e-16	0.5105	< 2.2e-16	< 2.2e-16	< 2.2e-16	< 2.2e-16
Edi-0	< 2.2e-16	< 2.2e-16	< 2.2e-16	< 2.2e-16	< 2.2e-16	< 2.2e-16
Hi-0	< 2.2e-16	< 2.2e-16	< 2.2e-16	< 2.2e-16	3.06E-11	< 2.2e-16
Kn-0	< 2.2e-16	< 2.2e-16	< 2.2e-16	< 2.2e-16	< 2.2e-16	< 2.2e-16
Ler-0	< 2.2e-16	< 2.2e-16	< 2.2e-16	< 2.2e-16	< 2.2e-16	< 2.2e-16
Mt-0	< 2.2e-16	< 2.2e-16	3.51E-08	< 2.2e-16	0.9029	< 2.2e-16
No-0	< 2.2e-16	< 2.2e-16	< 2.2e-16	< 2.2e-16	< 2.2e-16	< 2.2e-16
Oy-0	< 2.2e-16	< 2.2e-16	< 2.2e-16	< 2.2e-16	< 2.2e-16	< 2.2e-16
Po-0	< 2.2e-16	< 2.2e-16	< 2.2e-16	< 2.2e-16	0.8177	< 2.2e-16
Rsch-4	< 2.2e-16	< 2.2e-16	< 2.2e-16	< 2.2e-16	< 2.2e-16	< 2.2e-16
Sf-2	< 2.2e-16	< 2.2e-16	< 2.2e-16	< 2.2e-16	0.004767	< 2.2e-16
Tsu-0	< 2.2e-16	< 2.2e-16	< 2.2e-16	< 2.2e-16	2.84E-06	< 2.2e-16
Wil-2	< 2.2e-16	< 2.2e-16	< 2.2e-16	0.3031	0.00428	0.0001447
Ws-0	< 2.2e-16	< 2.2e-16	< 2.2e-16	< 2.2e-16	0.005222	< 2.2e-16
Wu-0	< 2.2e-16	< 2.2e-16	< 2.2e-16	< 2.2e-16	0.004152	< 2.2e-16
Zu-0	< 2.2e-16	< 2.2e-16	1.35E-09	< 2.2e-16	< 2.2e-16	< 2.2e-16

se: sense

as:antisense

Bold: the accessions with the order of TDI not follow Seedling<Root <Flower

Table S10 Average TDI (*A. thaliana* vs. *B. rapa*) and standard error of 19 accessions done by bootstrap 1000 times. se: sense; as: antisense.

Strains	Seedling_se	Root_se	Flower_se	Seedling_as	Root_as	Flower_as
Bur-0	0.1375594±0.00006987966	0.1449039±0.00007607015	0.1511653±0.0001070071	0.2242835±0.0004052374	0.2174545±0.0002569827	0.2132225±0.0001718478
Can-0	0.1392578±0.00007039052	0.1457511±0.00007481674	0.1590501±0.0001049946	0.2134961±0.0002086454	0.2103281±0.0001734902	0.208327±0.0002035035
Col-0	0.1360302±0.00007580117	0.1445145±0.00007084268	0.1584294±0.0001076169	0.2244535±0.0003003255	0.2300378±0.0004111513	0.2165742±0.0002509853
**Ct-1**	**0.1415464±0.00007740008**	**0.1415012±0.00007027343**	**0.1596407±0.00009445225**	0.2188311±0.0001831396	0.2237295±0.0001866008	0.2141812±0.0001581992
Edi-0	0.1412775±0.0000876645	0.1436163±0.00007625086	0.1598364±0.00008768771	0.2210404±0.000176926	0.2409652±0.0006327357	0.2155666±0.0001953092
Hi-0	0.137918±0.00007242653	0.1435598±0.00006884899	0.1620977±0.00009839111	0.2413827±0.0006242669	0.2402703±0.0006234554	0.225741±0.0003843577
Kn-0	0.1354982±0.0000775869	0.1417227±0.00006726478	0.1560322±0.0000771585	0.2229251±0.0002169544	0.2171072±0.0001473891	0.216958±0.0002181833
Ler-0	0.1325106±0.00007622343	0.1490607±0.00009385896	0.1593183±0.00009878341	0.2276146±0.0003409847	0.2238064±0.0002098437	0.217117±0.0002602656
Mt-0	0.1294945±0.0001777657	0.1399368±0.0001497379	0.1435551±0.0004700731	0.2319528±0.0005241576	0.2283151±0.0004682848	0.2095142±0.0002505997
No-0	0.1353337±0.00008506213	0.1424212±0.00006717332	0.1592437±0.00008316291	0.2330321±0.0004623435	0.212362±0.0001743249	0.2093403±0.000251225
Oy-0	0.1343592±0.00007512417	0.1454639±0.00007135778	0.1662982±0.0001015705	0.2301989±0.0002838258	0.2194952±0.0002710635	0.2128348±0.0002060076
Po-0	0.142977±0.00008620182	0.1497542±0.00008031821	0.1608686±0.0001106258	0.2288165±0.0002919771	0.2171267±0.0002015567	0.2147898±0.0002127014
Rsch-4	0.1296688±0.0001423052	0.1325505±0.0001354499	0.143127±0.000478397	0.2194883±0.0001971774	0.2228991±0.0001660577	0.2120015±0.0001689357
**Sf-2**	**0.1414106±0.0001012142**	**0.1393361±0.00006449684**	**0.1645352±0.0001025135**	0.2220277±0.0003220896	0.223545±0.0003105255	0.2099278±0.0002612001
Tsu-0	0.1347357±0.00007598645	0.1414829±0.00006653964	0.1609321±0.00008598075	0.2237539±0.0002788602	0.2203633±0.0001737286	0.2159888±0.0002027093
**Wil-2**	**0.1264022±0.0001593442**	**0.1405858±0.0001353452**	**0.1371348±0.0004863689**	0.2240413±0.0002699081	0.2224843±0.0002488465	0.2201113±0.0003124853
Ws-0	0.1352418±0.00007556519	0.1459548±0.00007136904	0.1563458±0.00009779759	0.2293352±0.0003769549	0.2331205±0.0004324116	0.219154±0.0002688134
Wu-0	0.1386614±0.00007716525	0.1445312±0.00006916723	0.1596704±0.0001067331	0.2186748±0.0002287848	0.2206974±0.0003693688	0.208164±0.0002373644
Zu-0	0.1301602±0.0002099897	0.1382043±0.0001323232	0.1400159±0.0004925649	0.2127825±0.0001390472	0.2149852±0.0001232411	0.2073411±0.0001058352

Table S11 P-value obtained from Mann-Whitney test for TDI for 19 accessions

Strains	Seedling_se vs. Flower_se	Seedling_se vs. Root_se	Root_se vs. Flower_se	Seedling_as vs. Flower_as	Seedling_as vs. Root_as	Root_as vs. Flower_as
Bur-0	< 2.2e-16	< 2.2e-16	< 2.2e-16	< 2.2e-16	< 2.2e-16	< 2.2e-16
Can-0	< 2.2e-16	< 2.2e-16	< 2.2e-16	< 2.2e-16	< 2.2e-16	< 2.2e-16
Col-0	< 2.2e-16	< 2.2e-16	< 2.2e-16	< 2.2e-16	< 2.2e-16	< 2.2e-16
Ct-1	< 2.2e-16	0.3858	< 2.2e-16	< 2.2e-16	< 2.2e-16	< 2.2e-16
Edi-0	< 2.2e-16	< 2.2e-16	< 2.2e-16	< 2.2e-16	< 2.2e-16	< 2.2e-16
Hi-0	< 2.2e-16	< 2.2e-16	< 2.2e-16	< 2.2e-16	0.1526	< 2.2e-16
Kn-0	< 2.2e-16	< 2.2e-16	< 2.2e-16	< 2.2e-16	< 2.2e-16	0.07718
Ler-0	< 2.2e-16	< 2.2e-16	< 2.2e-16	< 2.2e-16	2.162E-15	< 2.2e-16
Mt-0	< 2.2e-16	< 2.2e-16	5.715E-14	< 2.2e-16	0.000003051	< 2.2e-16
No-0	< 2.2e-16	< 2.2e-16	< 2.2e-16	< 2.2e-16	< 2.2e-16	< 2.2e-16
Oy-0	< 2.2e-16	< 2.2e-16	< 2.2e-16	< 2.2e-16	< 2.2e-16	< 2.2e-16
Po-0	< 2.2e-16	< 2.2e-16	< 2.2e-16	< 2.2e-16	< 2.2e-16	< 2.2e-16
Rsch-4	< 2.2e-16	< 2.2e-16	< 2.2e-16	< 2.2e-16	< 2.2e-16	< 2.2e-16
Sf-2	< 2.2e-16	< 2.2e-16	< 2.2e-16	< 2.2e-16	0.004767	< 2.2e-16
Tsu-0	< 2.2e-16	< 2.2e-16	< 2.2e-16	< 2.2e-16	< 2.2e-16	< 2.2e-16
Wil-2	< 2.2e-16	< 2.2e-16	1.973E-14	< 2.2e-16	0.00001843	9.434E-11
Ws-0	< 2.2e-16	< 2.2e-16	< 2.2e-16	< 2.2e-16	6.358E-09	< 2.2e-16
Wu-0	< 2.2e-16	< 2.2e-16	< 2.2e-16	< 2.2e-16	0.004152	< 2.2e-16
Zu-0	< 2.2e-16	< 2.2e-16	0.000104	< 2.2e-16	< 2.2e-16	< 2.2e-16

se: sense

as:antisense

Bold: the accessions with the order of TDI not follow Seedling<Root <Flower

Table S12 GO terms of Floral bud-specific sense expressed genes

GO term	Ontology	Description	Number in input list	Number in BG/Ref	P-value	FDR	-log(FDR)
GO:0035295	P	tube development	142	290	1.8E-85	3E-82	81.5228787
GO:0048868	P	pollen tube development	142	290	1.8E-85	3E-82	81.5228787
GO:0009860	P	pollen tube growth	130	248	2.1E-83	2.4E-80	79.6197888
GO:0009856	P	pollination	147	355	7.3E-76	6.1E-73	72.2146702
GO:0009827	P	plant-type cell wall modification	107	209	7.4E-68	5E-65	64.30103
GO:0009932	P	cell tip growth	130	334	1E-63	5.9E-61	60.229148
GO:0048610	P	reproductive cellular process	134	401	2.5E-56	1.2E-53	52.9208188
GO:0042545	P	cell wall modification	124	358	2.5E-54	1E-51	51
GO:0000904	P	cell morphogenesis involved in differentiation	130	394	4.9E-54	1.8E-51	50.7447275
GO:0048588	P	developmental cell growth	130	429	2.4E-49	8E-47	46.09691
GO:0009826	P	unidimensional cell growth	135	478	3E-47	8.5E-45	44.0705811
GO:0060560	P	developmental growth involved in morphogene	135	478	3E-47	8.5E-45	44.0705811
GO:0009664	P	plant-type cell wall organization	107	352	2.6E-41	6.8E-39	38.1674911
GO:0005576	C	extracellular region	309	1976	4.9E-41	1.5E-38	37.8239087
GO:0032989	P	cellular component morphogenesis	153	698	1.5E-38	3.5E-36	35.455932
GO:0071669	P	plant-type cell wall organization or biogenesis	114	451	1.9E-35	4.2E-33	32.3767507
GO:0000902	P	cell morphogenesis	136	661	1.1E-31	2.3E-29	28.6382722
GO:0030599	F	pectinesterase activity	52	109	3.3E-32	3.3E-29	28.4814861
GO:0016049	P	cell growth	138	686	4.5E-31	8.8E-29	28.0555173
GO:0071555	P	cell wall organization	124	578	6.2E-31	1.2E-28	27.9208188
GO:0008361	P	regulation of cell size	140	708	1.1E-30	1.9E-28	27.7212464
GO:0032535	P	regulation of cellular component size	140	720	6.4E-30	1E-27	27
GO:0090066	P	regulation of anatomical structure size	140	720	6.4E-30	1E-27	27
GO:0048468	P	cell development	133	681	9.1E-29	1.4E-26	25.853872
GO:0048589	P	developmental growth	139	759	4.5E-27	6.6E-25	24.1804561
GO:0040007	P	growth	144	909	9.3E-22	1.3E-19	18.8860566
GO:0004091	F	carboxylesterase activity	70	281	2.6E-22	1.3E-19	18.8860566
GO:0004857	F	enzyme inhibitor activity	47	140	2.3E-21	7.7E-19	18.1135093
GO:0046910	F	pectinesterase inhibitor activity	28	49	6.4E-21	1.6E-18	17.79588
GO:0048869	P	cellular developmental process	181	1373	4.8E-18	6.4E-16	15.19382
GO:0030154	P	cell differentiation	153	1090	5E-18	6.6E-16	15.1804561

GO:0071554	P	cell wall organization or biogenesis	133	919	4.8E-17	5.9E-15	14.229148
GO:0030705	P	cytoskeleton-dependent intracellular transport	30	77	2.7E-16	3.2E-14	13.49485
GO:0030048	P	actin filament-based movement	30	77	2.7E-16	3.2E-14	13.49485
GO:0000003	P	reproduction	243	2108	3.4E-16	3.8E-14	13.4202164
GO:0022414	P	reproductive process	235	2062	3E-15	3.2E-13	12.49485
GO:0010584	P	pollen exine formation	21	40	4.7E-15	5E-13	12.30103
GO:0010927	P	cellular component assembly involved in morp	21	42	1.7E-14	1.7E-12	11.7695511
GO:0010208	P	pollen wall assembly	21	42	1.7E-14	1.7E-12	11.7695511
GO:0045229	P	external encapsulating structure organization	22	58	4.4E-12	4.3E-10	9.36653154
GO:0009555	P	pollen development	53	284	5.7E-12	5.3E-10	9.27572413
GO:0009653	P	anatomical structure morphogenesis	186	1665	6E-12	5.5E-10	9.25963731
GO:0003006	P	reproductive developmental process	206	1904	9.6E-12	8.6E-10	9.06550155
GO:0030234	F	enzyme regulator activity	55	321	7.1E-11	1.4E-08	7.85387196
GO:0048235	P	pollen sperm cell differentiation	13	26	1.7E-09	0.00000015	6.82390874
GO:0005451	F	monovalent cation:hydrogen antiporter activity	15	36	2.3E-09	0.00000039	6.40893539
GO:0015299	F	solute:hydrogen antiporter activity	16	42	3.3E-09	0.00000047	6.32790214
GO:0015385	F	sodium:hydrogen antiporter activity	13	28	5.4E-09	0.00000068	6.16749109
GO:0048465	P	corolla development	25	107	1.9E-08	0.0000015	5.82390874
GO:0048441	P	petal development	25	107	1.9E-08	0.0000015	5.82390874
GO:0048232	P	male gamete generation	14	36	2.3E-08	0.0000018	5.74472749
GO:0004553	F	hydrolase activity, hydrolyzing O-glycosyl con	52	344	1.8E-08	0.0000021	5.67778071
GO:0015300	F	solute:solute antiporter activity	19	71	0.00000009	0.0000091	5.04095861
GO:0015298	F	solute:cation antiporter activity	16	52	0.00000011	0.00001	5
GO:0016798	F	hydrolase activity, acting on glycosyl bonds	52	368	0.00000016	0.000014	4.85387196
GO:0016788	F	hydrolase activity, acting on ester bonds	101	909	0.00000024	0.000019	4.7212464
GO:0060284	P	regulation of cell development	9	17	0.00000031	0.000022	4.65757732
GO:0010769	P	regulation of cell morphogenesis involved in d	9	17	0.00000031	0.000022	4.65757732
GO:0055046	P	microgametogenesis	13	37	0.00000029	0.000022	4.65757732
GO:0080092	P	regulation of pollen tube growth	9	17	0.00000031	0.000022	4.65757732
GO:0051704	P	multi-organism process	169	1729	0.00000034	0.000024	4.61978876
GO:0012511	C	monolayer-surrounded lipid storage body	8	13	0.0000003	0.000031	4.50863831
GO:0005811	C	lipid particle	8	13	0.0000003	0.000031	4.50863831
GO:0015491	F	cation:cation antiporter activity	15	51	0.00000052	0.000037	4.43179828

GO:0065008	P	regulation of biological quality	155	1578	0.0000007	0.000049	4.30980392
GO:0004650	F	polygalacturonase activity	14	49	0.0000018	0.00012	3.92081875
GO:0048466	P	androecium development	28	163	0.0000024	0.00016	3.79588002
GO:0048443	P	stamen development	28	163	0.0000024	0.00016	3.79588002
GO:0015297	F	antiporter activity	24	130	0.0000034	0.00021	3.67778071
GO:0048229	P	gametophyte development	59	488	0.0000048	0.00032	3.49485002
GO:0048440	P	carpel development	34	227	0.0000054	0.00035	3.45593196
GO:0019953	P	sexual reproduction	35	238	0.000006	0.00038	3.4202164
GO:0007626	P	locomotory behavior	7	14	0.000011	0.00064	3.19382003
GO:0010183	P	pollen tube guidance	7	14	0.000011	0.00064	3.19382003
GO:0006935	P	chemotaxis	7	14	0.000011	0.00064	3.19382003
GO:0050918	P	positive chemotaxis	7	14	0.000011	0.00064	3.19382003
GO:0042330	P	taxis	7	14	0.000011	0.00064	3.19382003
GO:0048467	P	gynoecium development	34	237	0.000014	0.00079	3.10237291
GO:0048646	P	anatomical structure formation involved in mor	40	302	0.000018	0.001	3
GO:0030029	P	actin filament-based process	35	251	0.000019	0.0011	2.95860731
GO:0048438	P	floral whorl development	54	457	0.000022	0.0012	2.92081875
GO:0007610	P	behavior	7	16	0.000033	0.0017	2.76955108
GO:0009567	P	double fertilization forming a zygote and endos	9	28	0.000045	0.0024	2.61978876
GO:0009566	P	fertilization	9	29	0.000061	0.0032	2.49485002
GO:0048856	P	anatomical structure development	264	3156	0.000062	0.0032	2.49485002
GO:0015291	F	secondary active transmembrane transporter ac	35	269	0.000081	0.0048	2.31875876
GO:0031224	C	intrinsic to membrane	101	1046	0.000078	0.005	2.30103
GO:0045177	C	apical part of cell	7	18	0.000081	0.005	2.30103
GO:0010092	P	specification of organ identity	9	31	0.00011	0.0054	2.26760624
GO:0010093	P	specification of floral organ identity	9	31	0.00011	0.0054	2.26760624
GO:0048445	P	carpel morphogenesis	6	14	0.00014	0.0069	2.16115091
GO:0017171	F	serine hydrolase activity	23	156	0.0002	0.011	1.95860731
GO:0008236	F	serine-type peptidase activity	23	156	0.0002	0.011	1.95860731
GO:0016324	C	apical plasma membrane	6	15	0.00022	0.011	1.95860731
GO:0032502	P	developmental process	305	3799	0.00029	0.014	1.85387196
GO:0040011	P	locomotion	7	22	0.00034	0.016	1.79588002
GO:0007275	P	multicellular organismal development	289	3597	0.00037	0.017	1.76955108

GO:0022604	P	regulation of cell morphogenesis	16	95	0.0004	0.018	1.74472749
GO:0015078	F	hydrogen ion transmembrane transporter activi	17	104	0.00038	0.019	1.7212464
GO:0048569	P	post-embryonic organ development	57	550	0.00043	0.02	1.69897
GO:0016787	F	hydrolase activity	224	2723	0.00042	0.02	1.69897
GO:0016298	F	lipase activity	18	116	0.0005	0.023	1.63827216
GO:0010333	F	terpene synthase activity	7	24	0.00062	0.027	1.56863624
GO:0022603	P	regulation of anatomical structure morphogene	18	118	0.00062	0.028	1.55284197
GO:0048437	P	floral organ development	56	547	0.00064	0.028	1.55284197
GO:0051510	P	regulation of unidimensional cell growth	15	91	0.00075	0.033	1.48148606
GO:0031225	C	anchored to membrane	26	207	0.001	0.045	1.34678749

Table S13 GO terms of Root-specific sense expressed genes

GO term	Ontology	Description	Number in input list	Number in BG/Ref	P-value	FDR	-log(FDR)
GO:0047134	F	protein-disulfide reductase activity	76	115	8.8E-60	8.4E-57	56.0757207
GO:0016668	F	oxidoreductase activity, acting on sulfur group of donors, NAD or NADP as acceptor	76	125	9.3E-56	4.4E-53	52.3565473
GO:0000041	P	transition metal ion transport	95	231	5.7E-49	2.1E-45	44.6777807
GO:0016651	F	oxidoreductase activity, acting on NADH or NADPH	80	189	9.2E-43	2.9E-40	39.537602
GO:0055114	P	oxidation reduction	209	1161	2.3E-36	4.4E-33	32.3565473
GO:0010106	P	cellular response to iron ion starvation	57	114	5.8E-36	7.2E-33	32.1426675
GO:0016667	F	oxidoreductase activity, acting on sulfur group of donors	82	249	2.6E-34	6.3E-32	31.2006595
GO:0016491	F	oxidoreductase activity	229	1465	2.7E-30	5.1E-28	27.2924298
GO:0006826	P	iron ion transport	53	123	1.6E-29	1.5E-26	25.8239087
GO:0010167	P	response to nitrate	60	195	6E-24	4.5E-21	20.3467875
GO:0005576	C	extracellular region	262	1976	1.6E-23	4.6E-21	20.3372422
GO:0020037	F	heme binding	76	318	3.2E-22	5E-20	19.30103
GO:0030001	P	metal ion transport	106	545	1.6E-22	9.7E-20	19.0132283
GO:0015706	P	nitrate transport	59	203	3.4E-22	1.8E-19	18.7447275
GO:0015698	P	inorganic anion transport	67	260	9.8E-22	4.6E-19	18.3372422
GO:0046906	F	tetrapyrrole binding	76	344	4.5E-20	6.1E-18	17.2146702
GO:0006820	P	anion transport	76	342	3.1E-20	1.3E-17	16.8860566
GO:0010054	P	trichoblast differentiation	74	334	1.1E-19	4.2E-17	16.3767507
GO:0009267	P	cellular response to starvation	68	296	4E-19	1.4E-16	15.853872
GO:0010053	P	root epidermal cell differentiation	74	345	7.8E-19	2.4E-16	15.6197888
GO:0005506	F	iron ion binding	84	433	2.9E-18	3.5E-16	15.455932
GO:0042594	P	response to starvation	68	302	1.2E-18	3.6E-16	15.4436975
GO:0031669	P	cellular response to nutrient levels	68	308	3.7E-18	9.9E-16	15.0043648
GO:0010015	P	root morphogenesis	82	431	2.3E-17	5.8E-15	14.236572
GO:0031667	P	response to nutrient levels	68	322	4.2E-17	9.8E-15	14.0087739
GO:0016705	F	oxidoreductase activity, acting on paired donors, with incorporation or reduction of molecular oxygen	73	383	1E-15	1.1E-13	12.9586073
GO:0048364	P	root development	96	578	5.7E-16	1.3E-13	12.8860566
GO:0022622	P	root system development	96	579	6.4E-16	1.3E-13	12.8860566
GO:0031668	P	cellular response to extracellular stimulus	68	345	1.6E-15	3.1E-13	12.5086383
GO:0071496	P	cellular response to external stimulus	68	346	1.8E-15	3.5E-13	12.455932
GO:0016684	F	oxidoreductase activity, acting on peroxide as acceptor	34	105	4.4E-15	3.8E-13	12.4202164

GO:0004601 F	peroxidase activity	34	105	4.4E-15	3.8E-13	12.4202164
GO:0009991 P	response to extracellular stimulus	68	360	1.4E-14	2.5E-12	11.60206
GO:0006812 P	cation transport	109	757	1.4E-13	2.4E-11	10.6197888
GO:0009913 P	epidermal cell differentiation	76	461	6.6E-13	1.1E-10	9.95860731
GO:0008544 P	epidermis development	76	463	8.3E-13	1.2E-10	9.92081875
GO:0007398 P	ectoderm development	76	463	8.3E-13	1.2E-10	9.92081875
GO:0016209 F	antioxidant activity	35	133	1.7E-12	1.3E-10	9.88605665
GO:0006811 P	ion transport	124	956	5.3E-12	7.7E-10	9.11350927
GO:0048765 P	root hair cell differentiation	56	304	6.9E-12	9.2E-10	9.03621217
GO:0048764 P	trichoblast maturation	56	304	6.9E-12	9.2E-10	9.03621217
GO:0048469 P	cell maturation	56	305	7.9E-12	1E-09	9
GO:0021700 P	developmental maturation	56	314	2.5E-11	3.2E-09	8.49485002
GO:0019825 F	oxygen binding	39	196	8.9E-10	6.5E-08	7.18708664
GO:0015674 P	di-, tri-valent inorganic cation transport	53	316	8.1E-10	9.8E-08	7.00877392
GO:0005199 F	structural constituent of cell wall	14	30	1.8E-09	1.2E-07	6.92081875
GO:0009055 F	electron carrier activity	63	436	1E-08	6.6E-07	6.18045606
GO:0043167 F	ion binding	239	2561	6.2E-07	3.5E-05	4.45593196
GO:0043169 F	cation binding	239	2561	6.2E-07	3.5E-05	4.45593196
GO:0046872 F	metal ion binding	226	2421	1.1E-06	5.9E-05	4.22914799
GO:0046914 F	transition metal ion binding	187	1944	1.2E-06	6.2E-05	4.20760831
GO:0010043 P	response to zinc ion	16	59	0.000001	0.00012	3.92081875
GO:0007043 P	cell-cell junction assembly	5	5	1.3E-06	0.00015	3.82390874
GO:0048226 C	Casparian strip	5	5	1.3E-06	0.00018	3.74472749
GO:0016706 F	oxidoreductase activity, acting on paired donors, with incorporation or reduction of molecular oxygen, 2-oxoglutarate as one donor, and incorporation of one atom each of oxygen into both donors	20	98	6.4E-06	0.0003	3.52287875
GO:0048527 P	lateral root development	23	118	3.1E-06	0.00034	3.46852108
GO:0048528 P	post-embryonic root development	24	127	3.4E-06	0.00036	3.4436975
GO:0043531 F	ADP binding	26	152	9.1E-06	0.00041	3.38721614
GO:0010333 F	terpene synthase activity	9	24	0.000013	0.00057	3.24412514
GO:0009691 P	cytokinin biosynthetic process	11	33	5.5E-06	0.00058	3.23657201
GO:0044426 C	cell wall part	5	6	7.3E-06	0.00069	3.16115091
GO:0034329 P	cell junction assembly	5	6	7.3E-06	0.00074	3.13076828
GO:0044462 C	external encapsulating structure part	5	7	0.000024	0.0017	2.76955108
GO:0016838 F	carbon-oxygen lyase activity, acting on phosphates	9	28	0.000055	0.0022	2.65757732
GO:0016765 F	transferase activity, transferring alkyl or aryl (other than methyl) groups	20	113	0.000056	0.0022	2.65757732
GO:0045216 P	cell-cell junction organization	5	7	0.000024	0.0024	2.61978876
GO:0032412 P	regulation of ion transmembrane transporter activity	10	32	0.000028	0.0024	2.61978876

GO:0010359	P	regulation of anion channel activity	10	32	0.000028	0.0024	2.61978876
GO:0009888	P	tissue development	100	981	0.000028	0.0024	2.61978876
GO:0022898	P	regulation of transmembrane transporter activity	10	32	0.000028	0.0024	2.61978876
GO:0032409	P	regulation of transporter activity	10	32	0.000028	0.0024	2.61978876
GO:0034765	P	regulation of ion transmembrane transport	10	32	0.000028	0.0024	2.61978876
GO:0034762	P	regulation of transmembrane transport	10	32	0.000028	0.0024	2.61978876
GO:0044421	C	extracellular region part	12	48	0.000055	0.0025	2.60205999
GO:0044420	C	extracellular matrix part	5	8	0.000061	0.0025	2.60205999
GO:0009531	C	secondary cell wall	5	8	0.000061	0.0025	2.60205999
GO:0007154	P	cell communication	75	690	0.000032	0.0026	2.58502665
GO:0034330	P	cell junction organization	5	8	0.000061	0.0049	2.30980392
GO:0003700	F	transcription factor activity	127	1382	0.00027	0.01	2
GO:0044070	P	regulation of anion transport	10	39	0.00018	0.014	1.85387196
GO:0005615	C	extracellular space	6	16	0.00039	0.014	1.85387196
GO:0010035	P	response to inorganic substance	101	1047	0.0002	0.015	1.82390874
GO:0008083	F	growth factor activity	5	11	0.00043	0.016	1.79588002
GO:0046274	P	lignin catabolic process	6	15	0.00026	0.019	1.7212464
GO:0046271	P	phenylpropanoid catabolic process	6	15	0.00026	0.019	1.7212464
GO:0030528	F	transcription regulator activity	129	1434	0.00055	0.019	1.7212464
GO:0048196	C	middle lamella-containing extracellular matrix	5	12	0.00069	0.022	1.65757732
GO:0004364	F	glutathione transferase activity	10	46	0.00075	0.025	1.60205999
GO:0009808	P	lignin metabolic process	13	67	0.00042	0.03	1.52287875
GO:0004568	F	chitinase activity	6	20	0.0015	0.047	1.32790214
GO:0045735	F	nutrient reservoir activity	8	34	0.0014	0.047	1.32790214

Table S14 GO terms for genes sense expressed in all three organs

GO term	Ontology	Description	Number in input list	Number in BG/Ref	P-value	FDR	-log(FDR)
GO:0044446	C	intracellular organelle part	2980	3156	1E-136	1.2E-133	132.920819
GO:0044422	C	organelle part	2984	3160	5.3E-137	1.2E-133	132.920819
GO:0006996	P	organelle organization	1904	1997	4E-103	6.9E-99	98.1611509
GO:0044444	C	cytoplasmic part	7274	8334	5.6E-87	4.4E-84	83.3565473
GO:0005829	C	cytosol	1531	1602	8E-87	4.7E-84	83.3279021
GO:0032991	C	macromolecular complex	1659	1777	1.7E-69	7.8E-67	66.1079054
GO:0005737	C	cytoplasm	8680	10164	9.8E-64	3.9E-61	60.4089354
GO:0044281	P	small molecule metabolic process	3414	3853	4.4E-62	3.8E-58	57.4202164
GO:0044267	P	cellular protein metabolic process	3320	3749	4.3E-60	2.5E-56	55.60206
GO:0006396	P	RNA processing	838	869	3.8E-55	1.6E-51	50.79588
GO:0019538	P	protein metabolic process	3605	4112	2.4E-54	8.4E-51	50.0757207
GO:0043232	C	intracellular non-membrane-bounded organelle	950	999	2.9E-52	8.5E-50	49.0705811
GO:0043228	C	non-membrane-bounded organelle	950	999	2.9E-52	8.5E-50	49.0705811
GO:0031090	C	organelle membrane	1151	1230	3.9E-51	1E-48	48
GO:0044237	P	cellular metabolic process	8217	9709	4.6E-50	1.3E-46	45.8860566
GO:0032259	P	methylation	530	536	4.3E-49	1.1E-45	44.9586073
GO:0043414	P	macromolecule methylation	522	528	2.9E-48	6.2E-45	44.2076083
GO:0044248	P	cellular catabolic process	1787	1977	6.3E-48	1.2E-44	43.9208188
GO:0009057	P	macromolecule catabolic process	1107	1186	9.5E-48	1.7E-44	43.7695511
GO:0044260	P	cellular macromolecule metabolic process	6009	7051	6.8E-47	1.1E-43	42.9586073
GO:0009056	P	catabolic process	1855	2062	3E-46	4.4E-43	42.3565473
GO:0006259	P	DNA metabolic process	712	740	5.3E-46	7.1E-43	42.1487417
GO:0051276	P	chromosome organization	699	726	1.4E-45	1.7E-42	41.7695511
GO:0044265	P	cellular macromolecule catabolic process	1062	1139	2.5E-45	2.9E-42	41.537602
GO:0009536	C	plastid	3127	3584	2.3E-44	5.4E-42	41.2676062
GO:0044435	C	plastid part	1082	1165	7.2E-44	1.5E-41	40.8239087
GO:0043234	C	protein complex	1144	1237	1E-43	2E-41	40.69897
GO:0006730	P	one-carbon metabolic process	550	562	2E-44	2.2E-41	40.6575773

GO:0009628	P	response to abiotic stimulus	2257	2545	3.6E-44	3.7E-41	40.4317983
GO:0044428	C	nuclear part	595	613	3.2E-43	5.9E-41	40.229148
GO:0007049	P	cell cycle	736	770	8.3E-44	8.1E-41	40.091515
GO:0009507	C	chloroplast	3063	3516	1.9E-42	3.3E-40	39.4814861
GO:0044434	C	chloroplast part	1061	1144	2.6E-42	4.1E-40	39.3872161
GO:0031967	C	organelle envelope	813	860	5.1E-42	7.1E-40	39.1487417
GO:0031975	C	envelope	813	860	5.1E-42	7.1E-40	39.1487417
GO:0006807	P	nitrogen compound metabolic process	4307	5015	1E-42	9.4E-40	39.0268721
GO:0043170	P	macromolecule metabolic process	6386	7542	2.1E-42	1.8E-39	38.7447275
GO:0022402	P	cell cycle process	548	564	1.8E-40	1.5E-37	36.8239087
GO:0016043	P	cellular component organization	2619	2992	1.9E-40	1.5E-37	36.8239087
GO:0043229	C	intracellular organelle	11823	14222	1.2E-39	1.6E-37	36.79588
GO:0043226	C	organelle	11826	14226	1.3E-39	1.6E-37	36.79588
GO:0051649	P	establishment of localization in cell	1364	1503	8.7E-40	6.6E-37	36.1804561
GO:0051641	P	cellular localization	1410	1559	4.5E-39	3.3E-36	35.4814861
GO:0046907	P	intracellular transport	1254	1378	2.7E-38	1.9E-35	34.7212464
GO:0043227	C	membrane-bounded organelle	11634	14020	3E-37	3.5E-35	34.455932
GO:0016568	P	chromatin modification	433	440	5.7E-38	3.8E-35	34.4202164
GO:0043231	C	intracellular membrane-bounded organelle	11629	14015	3.6E-37	4.1E-35	34.3872161
GO:0006412	P	translation	544	563	1.4E-37	9.2E-35	34.0362122
GO:0006325	P	chromatin organization	534	553	1.2E-36	7.6E-34	33.1191864
GO:0044424	C	intracellular part	13349	16162	5.6E-35	6E-33	32.2218487
GO:0005622	C	intracellular	13407	16235	7.1E-35	7.3E-33	32.1366771
GO:0009058	P	biosynthetic process	5546	6570	1.3E-35	8E-33	32.09691
GO:0044238	P	primary metabolic process	8193	9814	2E-35	1.2E-32	31.9208188
GO:0044249	P	cellular biosynthetic process	5362	6348	3E-35	1.7E-32	31.7695511
GO:0016569	P	covalent chromatin modification	405	412	4E-35	2.1E-32	31.6777807
GO:0044262	P	cellular carbohydrate metabolic process	1543	1728	3.9E-35	2.1E-32	31.6777807
GO:0006066	P	alcohol metabolic process	1021	1115	1.5E-34	7.5E-32	31.1249387
GO:0006139	P	nucleobase, nucleoside, nucleotide and nucleic acid metabolic process	3579	4181	1.8E-34	8.8E-32	31.0555173
GO:0044271	P	cellular nitrogen compound biosynthetic process	733	782	2.3E-34	1.1E-31	30.9586073
GO:0005996	P	monosaccharide metabolic process	737	787	3.8E-34	1.8E-31	30.7447275
GO:0015031	P	protein transport	1023	1120	1.8E-33	7.8E-31	30.1079054
GO:0008104	P	protein localization	1070	1175	1.8E-33	7.8E-31	30.1079054
GO:0045184	P	establishment of protein localization	1023	1120	1.8E-33	7.8E-31	30.1079054
GO:0000278	P	mitotic cell cycle	297	297	2.1E-33	9.1E-31	30.0409586
GO:0034613	P	cellular protein localization	941	1026	9.1E-33	3.8E-30	29.4202164

GO:0006886	P	intracellular protein transport	928	1011	1.1E-32	4.3E-30	29.3665315
GO:0034641	P	cellular nitrogen compound metabolic process	1181	1308	1.6E-32	6.3E-30	29.2006595
GO:0006605	P	protein targeting	814	879	1.6E-32	6.3E-30	29.2006595
GO:0005515	F	protein binding	2177	2491	1.3E-33	8.3E-30	29.0809219
GO:0043412	P	macromolecule modification	2549	2945	2.5E-32	9.6E-30	29.0177288
GO:0009532	C	plastid stroma	558	587	1.3E-31	1.2E-29	28.9208188
GO:0070727	P	cellular macromolecule localization	966	1057	5.1E-32	1.9E-29	28.7212464
GO:0009570	C	chloroplast stroma	544	572	4.6E-31	4.3E-29	28.3665315
GO:0008652	P	cellular amino acid biosynthetic process	477	496	2.3E-31	8.2E-29	28.0861861
GO:0006260	P	DNA replication	322	325	2.6E-31	9.2E-29	28.0362122
GO:0040029	P	regulation of gene expression, epigenetic	429	443	7.4E-31	2.6E-28	27.5850267
GO:0030529	C	ribonucleoprotein complex	481	502	2.9E-30	2.6E-28	27.5850267
GO:0016458	P	gene silencing	379	388	1.8E-30	6E-28	27.2218487
GO:0043436	P	oxoacid metabolic process	1745	1986	2.3E-30	7.4E-28	27.1307683
GO:0019752	P	carboxylic acid metabolic process	1745	1986	2.3E-30	7.4E-28	27.1307683
GO:0016570	P	histone modification	357	364	2.8E-30	9E-28	27.0457575
GO:0006082	P	organic acid metabolic process	1746	1988	3.6E-30	1.1E-27	26.9586073
GO:0042180	P	cellular ketone metabolic process	1760	2005	4.1E-30	1.3E-27	26.8860566
GO:0030163	P	protein catabolic process	468	488	8.2E-30	2.5E-27	26.60206
GO:0031974	C	membrane-enclosed lumen	559	592	3.1E-29	2.7E-27	26.5686362
GO:0046686	P	response to cadmium ion	443	460	9.1E-30	2.8E-27	26.552842
GO:0070013	C	intracellular organelle lumen	553	586	9.8E-29	8E-27	26.09691
GO:0043233	C	organelle lumen	553	586	9.8E-29	8E-27	26.09691

GO:0044272	P	sulfur compound biosynthetic process	462	482	2.9E-29	8.5E-27	26.0705811
GO:0031981	C	nuclear lumen	431	448	1.2E-28	9.2E-27	26.0362122
GO:0003723	F	RNA binding	542	571	3E-30	9.3E-27	26.0315171
GO:0009309	P	amine biosynthetic process	491	515	4.5E-29	1.3E-26	25.8860566
GO:0003735	F	structural constituent of ribosome	341	347	1E-29	2.1E-26	25.6777807
GO:0005773	C	vacuole	762	828	3E-28	2.3E-26	25.6382722
GO:0006479	P	protein amino acid methylation	298	301	8.5E-29	2.4E-26	25.6197888
GO:0008213	P	protein amino acid alkylation	298	301	8.5E-29	2.4E-26	25.6197888
GO:0044257	P	cellular protein catabolic process	453	473	1.8E-28	5.1E-26	25.2924298
GO:0005840	C	ribosome	396	410	9.7E-28	7.1E-26	25.1487417
GO:0016571	P	histone methylation	289	292	7.5E-28	2E-25	24.69897
GO:0005794	C	Golgi apparatus	841	923	4.1E-27	3E-25	24.5228787
GO:0044106	P	cellular amine metabolic process	818	895	1.5E-27	4.1E-25	24.3872161
GO:0006520	P	cellular amino acid metabolic process	768	837	2.2E-27	5.9E-25	24.229148
GO:0017038	P	protein import	346	355	3.1E-27	8.1E-25	24.091515
GO:0005774	C	vacuolar membrane	445	467	2.3E-26	1.6E-24	23.79588
GO:0051603	P	proteolysis involved in cellular protein catabolic process	427	446	7.9E-27	2E-24	23.69897
GO:0009526	C	plastid envelope	534	569	5E-26	3.4E-24	23.4685211
GO:0044437	C	vacuolar part	447	470	6.9E-26	4.6E-24	23.3372422
GO:0044085	P	cellular component biogenesis	1182	1328	2.1E-26	5.3E-24	23.2757241
GO:0006464	P	protein modification process	2226	2584	2.1E-26	5.3E-24	23.2757241
GO:0031047	P	gene silencing by RNA	305	311	4.4E-26	1.1E-23	22.9586073
GO:0000097	P	sulfur amino acid biosynthetic process	303	309	7.1E-26	1.7E-23	22.7695511
GO:0044282	P	small molecule catabolic process	816	897	8.1E-26	1.9E-23	22.7212464
GO:0009941	C	chloroplast envelope	514	548	5.9E-25	3.8E-23	22.4202164
GO:0043933	P	macromolecular complex subunit organization	592	637	2.2E-25	5.2E-23	22.2839967
GO:0019318	P	hexose metabolic process	551	590	3.1E-25	7.2E-23	22.1426675
GO:0005911	C	cell-cell junction	749	822	2E-24	1.3E-22	21.8860566
GO:0006261	P	DNA-dependent DNA replication	249	251	6E-25	1.4E-22	21.853872
GO:0009506	C	plasmodesma	747	820	2.8E-24	1.6E-22	21.79588

GO:0055044	C	symplast	747	820	2.8E-24	1.6E-22	21.79588
GO:0006511	P	ubiquitin-dependent protein catabolic process	348	360	7.9E-25	1.8E-22	21.7447275
GO:0030054	C	cell junction	751	825	3.6E-24	2.1E-22	21.6777807
GO:0009987	P	cellular process	10062	12252	1E-24	2.3E-22	21.6382722
GO:0065003	P	macromolecular complex assembly	569	612	1.3E-24	2.9E-22	21.537602
GO:0008654	P	phospholipid biosynthetic process	376	392	2.3E-24	5.1E-22	21.2924298
GO:0043632	P	modification-dependent macromolecule catabolic process	351	364	2.6E-24	5.5E-22	21.2596373
GO:0019941	P	modification-dependent protein catabolic process	351	364	2.6E-24	5.5E-22	21.2596373
GO:0006753	P	nucleoside phosphate metabolic process	609	659	2.8E-24	5.9E-22	21.229148
GO:0051301	P	cell division	342	354	2.9E-24	6E-22	21.2218487
GO:0048193	P	Golgi vesicle transport	315	324	3.3E-24	6.8E-22	21.1674911
GO:0008380	P	RNA splicing	276	281	3.4E-24	7E-22	21.154902
GO:0009117	P	nucleotide metabolic process	607	657	3.9E-24	7.9E-22	21.1023729
GO:0009314	P	response to radiation	1084	1219	4E-24	7.9E-22	21.1023729
GO:0019637	P	organophosphate metabolic process	441	466	4.2E-24	8.2E-22	21.0861861
GO:0007010	P	cytoskeleton organization	469	498	4.4E-24	8.6E-22	21.0655015
GO:0006006	P	glucose metabolic process	509	544	5.2E-24	1E-21	21
GO:0055086	P	nucleobase, nucleoside and nucleotide metabolic process	635	690	6.3E-24	1.2E-21	20.9208188
GO:0034621	P	cellular macromolecular complex subunit organization	534	573	6.7E-24	1.3E-21	20.8860566
GO:0034968	P	histone lysine methylation	238	240	8.8E-24	1.6E-21	20.79588
GO:0051789	P	response to protein stimulus	345	358	9.3E-24	1.7E-21	20.7695511
GO:0016020	C	membrane	4645	5569	3.8E-23	2.1E-21	20.6777807
GO:0033205	P	cytokinesis during cell cycle	207	207	1.5E-23	2.7E-21	20.5686362
GO:0009165	P	nucleotide biosynthetic process	307	316	2E-23	3.6E-21	20.4436975
GO:0006644	P	phospholipid metabolic process	432	457	2.4E-23	4.3E-21	20.3665315
GO:0034622	P	cellular macromolecular complex assembly	512	549	3.4E-23	6E-21	20.2218487
GO:0006974	P	response to DNA damage stimulus	322	333	3.5E-23	6.1E-21	20.2146702
GO:0000911	P	cytokinesis by cell plate formation	203	203	4.1E-23	7.1E-21	20.1487417
GO:0000910	P	cytokinesis	231	233	4.9E-23	8.4E-21	20.0757207
GO:0033279	C	ribosomal subunit	249	253	1.6E-22	9E-21	20.0457575

GO:0007017	P	microtubule-based process	302	311	6E-23	1E-20	20
GO:0051726	P	regulation of cell cycle	280	287	1.3E-22	2.2E-20	19.6575773
GO:0006090	P	pyruvate metabolic process	372	390	1.3E-22	2.2E-20	19.6575773
GO:0022613	P	ribonucleoprotein complex biogenesis	363	380	1.8E-22	2.9E-20	19.537602
GO:0046483	P	heterocycle metabolic process	862	961	2.5E-22	4.1E-20	19.3872161
GO:0005730	C	nucleolus	281	289	8.7E-22	4.7E-20	19.3279021
GO:0051186	P	cofactor metabolic process	670	735	3.3E-22	5.4E-20	19.2676062
GO:0006461	P	protein complex assembly	492	528	3.9E-22	6.1E-20	19.2146702
GO:0070271	P	protein complex biogenesis	492	528	3.9E-22	6.1E-20	19.2146702
GO:0044445	C	cytosolic part	239	243	1.7E-21	9.1E-20	19.0409586
GO:0005768	C	endosome	268	275	2E-21	1.1E-19	18.9586073
GO:0046365	P	monosaccharide catabolic process	442	471	7.4E-22	1.2E-19	18.9208188
GO:0034660	P	ncRNA metabolic process	377	397	1E-21	1.6E-19	18.79588
GO:0042254	P	ribosome biogenesis	353	370	1.4E-21	2.1E-19	18.6777807
GO:0019320	P	hexose catabolic process	438	467	1.6E-21	2.4E-19	18.6197888
GO:0046164	P	alcohol catabolic process	451	482	1.7E-21	2.6E-19	18.5850267
GO:0005802	C	trans-Golgi network	223	226	5.8E-21	2.9E-19	18.537602
GO:0006342	P	chromatin silencing	238	242	2.2E-21	3.3E-19	18.4814861
GO:0009657	P	plastid organization	373	393	2.2E-21	3.3E-19	18.4814861
GO:0006007	P	glucose catabolic process	436	465	2.3E-21	3.3E-19	18.4814861
GO:0010467	P	gene expression	2897	3433	2.3E-21	3.3E-19	18.4814861
GO:0044459	C	plasma membrane part	907	1020	1.4E-20	6.7E-19	18.1739252
GO:0033036	P	macromolecule localization	1254	1434	4.7E-21	6.9E-19	18.1611509
GO:0005975	P	carbohydrate metabolic process	1820	2119	4.8E-21	6.9E-19	18.1611509
GO:0009416	P	response to light stimulus	1013	1145	5E-21	7.2E-19	18.1426675
GO:0009100	P	glycoprotein metabolic process	222	225	7.4E-21	1.1E-18	17.9586073
GO:0043623	P	cellular protein complex assembly	435	465	9.5E-21	1.3E-18	17.8860566
GO:0006281	P	DNA repair	279	288	9.9E-21	1.4E-18	17.853872
GO:0022403	P	cell cycle phase	320	334	1E-20	1.5E-18	17.8239087
GO:0070085	P	glycosylation	218	221	1.9E-20	2.6E-18	17.5850267

GO:0043413	P	macromolecule glycosylation	218	221	1.9E-20	2.6E-18	17.5850267
GO:0009101	P	glycoprotein biosynthetic process	218	221	1.9E-20	2.6E-18	17.5850267
GO:0006486	P	protein amino acid glycosylation	218	221	1.9E-20	2.6E-18	17.5850267
GO:0009308	P	amine metabolic process	867	973	2.1E-20	2.8E-18	17.552842
GO:0007030	P	Golgi organization	178	178	2.3E-20	3E-18	17.5228787
GO:0009892	P	negative regulation of metabolic process	541	589	3.4E-20	4.5E-18	17.3467875
GO:0044431	C	Golgi apparatus part	293	305	1.1E-19	5.2E-18	17.2839967
GO:0010608	P	posttranscriptional regulation of gene expression	271	280	5.7E-20	7.5E-18	17.1249387
GO:0005198	F	structural molecule activity	445	476	5.1E-21	7.9E-18	17.1023729
GO:0009059	P	macromolecule biosynthetic process	3410	4075	6.7E-20	8.6E-18	17.0655015
GO:0009651	P	response to salt stress	701	778	8.9E-20	1.1E-17	16.9586073
GO:0006970	P	response to osmotic stress	743	828	1.1E-19	1.4E-17	16.853872
GO:0045814	P	negative regulation of gene expression, epigenetic	241	247	1.1E-19	1.4E-17	16.853872
GO:0006096	P	glycolysis	210	213	1.3E-19	1.6E-17	16.79588
GO:0000226	P	microtubule cytoskeleton organization	240	246	1.4E-19	1.8E-17	16.7447275
GO:0005623	C	cell	14834	18297	5.8E-19	2.7E-17	16.5686362
GO:0044464	C	cell part	14834	18297	5.8E-19	2.7E-17	16.5686362
GO:0022626	C	cytosolic ribosome	277	288	5.9E-19	2.7E-17	16.5686362
GO:0016192	P	vesicle-mediated transport	528	576	3E-19	3.7E-17	16.4317983
GO:0006081	P	cellular aldehyde metabolic process	311	326	3.4E-19	4.1E-17	16.3872161
GO:0010228	P	vegetative to reproductive phase transition of meristem	402	430	3.6E-19	4.5E-17	16.3467875
GO:0010605	P	negative regulation of macromolecule metabolic process	520	567	4.1E-19	5E-17	16.30103
GO:0048519	P	negative regulation of biological process	1014	1154	6.5E-19	7.8E-17	16.1079054
GO:0033365	P	protein localization in organelle	251	259	7E-19	8.4E-17	16.0757207
GO:0005739	C	mitochondrion	2072	2442	2.3E-18	1E-16	16
GO:0051567	P	histone H3-K9 methylation	178	179	9.3E-19	1.1E-16	15.9586073
GO:0001510	P	RNA methylation	178	179	9.3E-19	1.1E-16	15.9586073
GO:0043687	P	post-translational protein modification	1656	1932	1.1E-18	1.3E-16	15.8860566
GO:0009790	P	embryonic development	537	588	1.2E-18	1.4E-16	15.853872
GO:0016051	P	carbohydrate biosynthetic process	916	1039	3.1E-18	3.6E-16	15.4436975

GO:0034645	P	cellular macromolecule biosynthetic process	3342	4006	3.9E-18	4.5E-16	15.3467875
GO:0022607	P	cellular component assembly	680	758	4.1E-18	4.6E-16	15.3372422
GO:0012505	C	endomembrane system	246	255	1.4E-17	5.9E-16	15.229148
GO:0006790	P	sulfur metabolic process	601	665	5.7E-18	6.5E-16	15.1870866
GO:0051052	P	regulation of DNA metabolic process	194	197	5.9E-18	6.7E-16	15.1739252
GO:0070647	P	protein modification by small protein conjugation or removal	367	392	6E-18	6.7E-16	15.1739252
GO:0016874	F	ligase activity	413	443	5.4E-19	6.7E-16	15.1739252
GO:0070646	P	protein modification by small protein removal	182	184	7.3E-18	8.1E-16	15.091515
GO:0016052	P	carbohydrate catabolic process	514	563	7.5E-18	8.3E-16	15.0809219
GO:0035194	P	posttranscriptional gene silencing by RNA	192	195	9.5E-18	1E-15	15
GO:0016246	P	RNA interference	180	182	1.2E-17	1.3E-15	14.8860566
GO:0009658	P	chloroplast organization	228	235	1.7E-17	1.8E-15	14.7447275
GO:0010629	P	negative regulation of gene expression	497	544	1.8E-17	1.9E-15	14.7212464
GO:0006534	P	cysteine metabolic process	208	213	2.5E-17	2.6E-15	14.5850267
GO:0005886	C	plasma membrane	2897	3467	6.6E-17	2.8E-15	14.552842
GO:0009069	P	serine family amino acid metabolic process	259	270	2.7E-17	2.9E-15	14.537602
GO:0019344	P	cysteine biosynthetic process	205	210	4.9E-17	5.2E-15	14.2839967
GO:0009793	P	embryonic development ending in seed dormancy	471	515	6.9E-17	7.3E-15	14.1366771
GO:0044275	P	cellular carbohydrate catabolic process	488	535	7.5E-17	7.9E-15	14.1023729
GO:0009259	P	ribonucleotide metabolic process	284	299	8.2E-17	8.5E-15	14.0705811
GO:0051788	P	response to misfolded protein	182	185	1E-16	1.1E-14	13.9586073
GO:0000279	P	M phase	275	289	1.1E-16	1.2E-14	13.9208188
GO:0010564	P	regulation of cell cycle process	142	142	2.1E-16	2.1E-14	13.6777807
GO:0032787	P	monocarboxylic acid metabolic process	1207	1398	2.3E-16	2.3E-14	13.6382722
GO:0016441	P	posttranscriptional gene silencing	198	203	2.4E-16	2.4E-14	13.6197888
GO:0019866	C	organelle inner membrane	235	245	8.1E-16	3.4E-14	13.4685211
GO:0009220	P	pyrimidine ribonucleotide biosynthetic process	140	140	3.4E-16	3.5E-14	13.455932
GO:0006519	P	cellular amino acid and derivative metabolic process	1079	1244	4E-16	4E-14	13.39794
GO:0009260	P	ribonucleotide biosynthetic process	204	210	4.9E-16	4.9E-14	13.3098039
GO:0034470	P	ncRNA processing	303	322	5.1E-16	5.1E-14	13.2924298

GO:0043248	P	proteasome assembly	175	178	5.4E-16	5.3E-14	13.2757241
GO:0044429	C	mitochondrial part	261	275	1.9E-15	8E-14	13.09691
GO:0006306	P	DNA methylation	173	176	8.6E-16	8.3E-14	13.0809219
GO:0006305	P	DNA alkylation	173	176	8.6E-16	8.3E-14	13.0809219
GO:0006275	P	regulation of DNA replication	135	135	1.2E-15	1.2E-13	12.9208188
GO:0051188	P	cofactor biosynthetic process	349	376	1.7E-15	1.6E-13	12.79588
GO:0045017	P	glycerolipid biosynthetic process	189	194	1.9E-15	1.8E-13	12.7447275
GO:0009070	P	serine family amino acid biosynthetic process	214	222	2.3E-15	2.2E-13	12.6575773
GO:0006091	P	generation of precursor metabolites and energy	538	598	2.5E-15	2.4E-13	12.6197888
GO:0009218	P	pyrimidine ribonucleotide metabolic process	146	147	2.5E-15	2.4E-13	12.6197888
GO:0043094	P	cellular metabolic compound salvage	178	182	2.7E-15	2.6E-13	12.5850267
GO:0048518	P	positive regulation of biological process	744	844	3.4E-15	3.1E-13	12.5086383
GO:0048522	P	positive regulation of cellular process	667	752	3.8E-15	3.5E-13	12.455932
GO:0048523	P	negative regulation of cellular process	677	764	3.9E-15	3.6E-13	12.4436975
GO:0006304	P	DNA modification	175	179	5.5E-15	5E-13	12.30103
GO:0046486	P	glycerolipid metabolic process	241	253	5.7E-15	5.2E-13	12.2839967
GO:0000096	P	sulfur amino acid metabolic process	413	452	7.5E-15	6.8E-13	12.1674911
GO:0046474	P	glycerophospholipid biosynthetic process	173	177	8.7E-15	7.8E-13	12.1079054
GO:0006220	P	pyrimidine nucleotide metabolic process	152	154	1E-14	9.1E-13	12.0409586
GO:0006650	P	glycerophospholipid metabolic process	223	233	1E-14	9.1E-13	12.0409586
GO:0034637	P	cellular carbohydrate biosynthetic process	799	912	1.1E-14	9.6E-13	12.0177288
GO:0007292	P	female gamete generation	140	141	1.1E-14	9.8E-13	12.0087739
GO:0009560	P	embryo sac egg cell differentiation	138	139	1.8E-14	1.6E-12	11.79588
GO:0009791	P	post-embryonic development	1719	2035	2.5E-14	2.2E-12	11.6575773
GO:0006221	P	pyrimidine nucleotide biosynthetic process	148	150	2.7E-14	2.3E-12	11.6382722
GO:0010154	P	fruit development	585	657	2.7E-14	2.3E-12	11.6382722
GO:0016818	F	hydrolase activity, acting on acid anhydrides, in phosphorus-containing anhydrides	720	815	2.9E-15	2.3E-12	11.6382722
GO:0016817	F	hydrolase activity, acting on acid anhydrides	730	827	2.9E-15	2.3E-12	11.6382722
GO:0016462	F	pyrophosphatase activity	711	804	2.5E-15	2.3E-12	11.6382722
GO:0044283	P	small molecule biosynthetic process	1495	1761	3.4E-14	2.9E-12	11.537602

GO:0009853	P	photorespiration	156	159	4.7E-14	4E-12	11.39794
GO:0015934	C	large ribosomal subunit	142	144	1.1E-13	4.6E-12	11.3372422
GO:0048608	P	reproductive structure development	1351	1586	5.6E-14	4.8E-12	11.3187588
GO:0006732	P	coenzyme metabolic process	445	492	6.2E-14	5.3E-12	11.2757241
GO:0006839	P	mitochondrial transport	144	146	6.9E-14	5.9E-12	11.229148
GO:0046394	P	carboxylic acid biosynthetic process	917	1058	7.3E-14	6.1E-12	11.2146702
GO:0016053	P	organic acid biosynthetic process	917	1058	7.3E-14	6.1E-12	11.2146702
GO:0048316	P	seed development	567	637	7.6E-14	6.3E-12	11.2006595
GO:0005783	C	endoplasmic reticulum	513	574	1.9E-13	7.7E-12	11.1135093
GO:0080008	C	CUL4 RING ubiquitin ligase complex	115	115	1.9E-13	7.7E-12	11.1135093
GO:0016070	P	RNA metabolic process	2655	3195	1E-13	8.3E-12	11.0809219
GO:0009240	P	isopentenyl diphosphate biosynthetic process	219	230	1.1E-13	9.2E-12	11.0362122
GO:0046490	P	isopentenyl diphosphate metabolic process	219	230	1.1E-13	9.2E-12	11.0362122
GO:0017111	F	nucleoside-triphosphatase activity	685	776	1.9E-14	1.3E-11	10.8860566
GO:0071359	P	cellular response to dsRNA	140	142	1.8E-13	1.5E-11	10.8239087
GO:0043331	P	response to dsRNA	140	142	1.8E-13	1.5E-11	10.8239087
GO:0031050	P	dsRNA fragmentation	140	142	1.8E-13	1.5E-11	10.8239087
GO:0070918	P	production of small RNA involved in gene silencing by RNA	140	142	1.8E-13	1.5E-11	10.8239087
GO:0051607	P	defense response to virus	150	153	1.9E-13	1.5E-11	10.8239087
GO:0006508	P	proteolysis	711	811	1.9E-13	1.5E-11	10.8239087
GO:0019682	P	glyceraldehyde-3-phosphate metabolic process	216	227	2.1E-13	1.6E-11	10.79588
GO:0000394	P	RNA splicing, via endonucleolytic cleavage and ligation	139	141	2.3E-13	1.8E-11	10.7447275
GO:0009266	P	response to temperature stimulus	792	909	2.3E-13	1.8E-11	10.7447275
GO:0031966	C	mitochondrial membrane	212	223	4.7E-13	1.8E-11	10.7447275
GO:0019288	P	isopentenyl diphosphate biosynthetic process, mevalonate-independent pathway	215	226	2.5E-13	2E-11	10.69897
GO:0016072	P	rRNA metabolic process	250	266	2.6E-13	2.1E-11	10.6777807
GO:0005740	C	mitochondrial envelope	225	238	6.1E-13	2.3E-11	10.6382722
GO:0006364	P	rRNA processing	249	265	3.2E-13	2.5E-11	10.60206
GO:0005634	C	nucleus	6300	7750	7.5E-13	2.8E-11	10.552842
GO:0031461	C	cullin-RING ubiquitin ligase complex	143	146	9.9E-13	3.6E-11	10.4436975

GO:0030422	P	production of siRNA involved in RNA interference	136	138	4.7E-13	3.7E-11	10.4317983
GO:0010038	P	response to metal ion	522	586	5E-13	3.8E-11	10.4202164
GO:0031324	P	negative regulation of cellular metabolic process	397	438	5.8E-13	4.5E-11	10.3467875
GO:0000166	F	nucleotide binding	2608	3136	8.2E-14	5.1E-11	10.2924298
GO:0010498	P	proteasomal protein catabolic process	195	204	7.2E-13	5.5E-11	10.2596373
GO:0006913	P	nucleocytoplasmic transport	193	202	1.1E-12	8.3E-11	10.0809219
GO:0051169	P	nuclear transport	193	202	1.1E-12	8.3E-11	10.0809219
GO:0051701	P	interaction with host	121	122	1.2E-12	9E-11	10.0457575
GO:0008283	P	cell proliferation	242	258	1.2E-12	9.2E-11	10.0362122
GO:0044451	C	nucleoplasm part	157	162	2.5E-12	9.3E-11	10.0315171
GO:0008152	P	metabolic process	9234	11432	1.4E-12	1.1E-10	9.95860731
GO:0007346	P	regulation of mitotic cell cycle	107	107	1.5E-12	1.1E-10	9.95860731
GO:0044255	P	cellular lipid metabolic process	1121	1313	1.6E-12	1.2E-10	9.92081875
GO:0016879	F	ligase activity, forming carbon-nitrogen bonds	315	341	2.3E-13	1.3E-10	9.88605665
GO:0009615	P	response to virus	175	182	1.8E-12	1.4E-10	9.85387196
GO:0006346	P	methylation-dependent chromatin silencing	116	117	4.1E-12	3E-10	9.52287875
GO:0005743	C	mitochondrial inner membrane	183	192	8.7E-12	3.1E-10	9.50863831
GO:0046364	P	monosaccharide biosynthetic process	178	186	5.1E-12	3.8E-10	9.4202164
GO:0035196	P	production of miRNAs involved in gene silencing by miRNA	126	128	5.2E-12	3.8E-10	9.4202164
GO:0052249	P	modulation of RNA levels in other organism during symbiotic interaction	102	102	5.3E-12	3.8E-10	9.4202164
GO:0052018	P	modulation by symbiont of RNA levels in host	102	102	5.3E-12	3.8E-10	9.4202164
GO:0009616	P	virus induced gene silencing	102	102	5.3E-12	3.8E-10	9.4202164
GO:0019319	P	hexose biosynthetic process	170	177	5.4E-12	3.9E-10	9.40893539
GO:0051239	P	regulation of multicellular organismal process	585	665	5.4E-12	3.9E-10	9.40893539
GO:0035195	P	gene silencing by miRNA	135	138	6.4E-12	4.5E-10	9.34678749
GO:0048580	P	regulation of post-embryonic development	392	435	6.7E-12	4.8E-10	9.31875876
GO:0010388	P	cullin deneddylation	101	101	6.8E-12	4.8E-10	9.31875876
GO:0000338	P	protein deneddylation	101	101	6.8E-12	4.8E-10	9.31875876
GO:0022625	C	cytosolic large ribosomal subunit	111	112	1.4E-11	4.9E-10	9.30980392
GO:0050793	P	regulation of developmental process	699	803	7.6E-12	5.3E-10	9.27572413

GO:0048285	P	organelle fission	133	136	1E-11	7E-10	9.15490196
GO:0006950	P	response to stress	3129	3804	1.1E-11	7.4E-10	9.13076828
GO:0000151	C	ubiquitin ligase complex	200	212	2.1E-11	7.4E-10	9.13076828
GO:0007005	P	mitochondrion organization	122	124	1.3E-11	9.2E-10	9.03621217
GO:0010558	P	negative regulation of macromolecule biosynthetic process	369	409	1.8E-11	1.2E-09	8.92081875
GO:0031327	P	negative regulation of cellular biosynthetic process	374	415	1.9E-11	1.3E-09	8.88605665
GO:0009890	P	negative regulation of biosynthetic process	374	415	1.9E-11	1.3E-09	8.88605665
GO:0010027	P	thylakoid membrane organization	186	196	2.1E-11	1.4E-09	8.85387196
GO:0009668	P	plastid membrane organization	186	196	2.1E-11	1.4E-09	8.85387196
GO:0051172	P	negative regulation of nitrogen compound metabolic process	367	407	2.4E-11	1.7E-09	8.76955108
GO:0044003	P	modification by symbiont of host morphology or physiology	108	109	2.9E-11	1.9E-09	8.7212464
GO:0051817	P	modification of morphology or physiology of other organism during symbiotic interaction	108	109	2.9E-11	1.9E-09	8.7212464
GO:0010267	P	production of ta-siRNAs involved in RNA interference	108	109	2.9E-11	1.9E-09	8.7212464
GO:0045934	P	negative regulation of nucleobase, nucleoside, nucleotide and nucleic acid metabolic process	365	405	3.3E-11	2.2E-09	8.65757732
GO:0006310	P	DNA recombination	224	240	3.6E-11	2.4E-09	8.61978876
GO:0046489	P	phosphoinositide biosynthetic process	107	108	3.7E-11	2.4E-09	8.61978876
GO:0005694	C	chromosome	180	190	7E-11	2.4E-09	8.61978876
GO:0004386	F	helicase activity	154	159	5E-12	2.6E-09	8.58502665
GO:0006401	P	RNA catabolic process	116	118	5.6E-11	3.7E-09	8.43179828
GO:0009648	P	photoperiodism	159	166	5.6E-11	3.7E-09	8.43179828
GO:0044419	P	interspecies interaction between organisms	125	128	6.4E-11	4.2E-09	8.37675071
GO:0009607	P	response to biotic stimulus	1350	1604	6.6E-11	4.3E-09	8.36653154
GO:0042023	P	DNA endoreduplication	115	117	7E-11	4.6E-09	8.33724217
GO:0006094	P	gluconeogenesis	157	164	8.5E-11	5.5E-09	8.25963731
GO:0006397	P	mRNA processing	149	155	8.9E-11	5.7E-09	8.24412514
GO:0006626	P	protein targeting to mitochondrion	103	104	9.9E-11	6.3E-09	8.20065945
GO:0070585	P	protein localization in mitochondrion	103	104	9.9E-11	6.3E-09	8.20065945
GO:0005654	C	nucleoplasm	168	177	1.9E-10	6.3E-09	8.20065945
GO:0051716	P	cellular response to stimulus	1819	2185	1E-10	6.4E-09	8.19382003
GO:0044403	P	symbiosis, encompassing mutualism through parasitism	123	126	1E-10	6.4E-09	8.19382003

GO:0080129	P	proteasome core complex assembly	123	126	1E-10	6.4E-09	8.19382003
GO:0046395	P	carboxylic acid catabolic process	351	390	1.2E-10	7.4E-09	8.13076828
GO:0016054	P	organic acid catabolic process	351	390	1.2E-10	7.4E-09	8.13076828
GO:0006399	P	tRNA metabolic process	131	135	1.2E-10	7.8E-09	8.1079054
GO:0009893	P	positive regulation of metabolic process	515	586	1.3E-10	8E-09	8.09691001
GO:0031325	P	positive regulation of cellular metabolic process	510	580	1.3E-10	8E-09	8.09691001
GO:0050896	P	response to stimulus	4736	5827	1.4E-10	8.5E-09	8.07058107
GO:0006635	P	fatty acid beta-oxidation	161	169	1.8E-10	1.1E-08	7.95860731
GO:0009891	P	positive regulation of biosynthetic process	497	565	1.9E-10	1.2E-08	7.92081875
GO:0031328	P	positive regulation of cellular biosynthetic process	497	565	1.9E-10	1.2E-08	7.92081875
GO:0043161	P	proteasomal ubiquitin-dependent protein catabolic process	120	123	2E-10	1.2E-08	7.92081875
GO:0007031	P	peroxisome organization	120	123	2E-10	1.2E-08	7.92081875
GO:0009086	P	methionine biosynthetic process	100	101	2.1E-10	1.2E-08	7.92081875
GO:0016481	P	negative regulation of transcription	352	392	2.3E-10	1.4E-08	7.85387196
GO:0009639	P	response to red or far red light	352	392	2.3E-10	1.4E-08	7.85387196
GO:0045892	P	negative regulation of transcription, DNA-dependent	352	392	2.3E-10	1.4E-08	7.85387196
GO:0051253	P	negative regulation of RNA metabolic process	352	392	2.3E-10	1.4E-08	7.85387196
GO:0048573	P	photoperiodism, flowering	152	159	2.4E-10	1.5E-08	7.82390874
GO:0015935	C	small ribosomal subunit	107	109	4.7E-10	1.6E-08	7.79588002
GO:0009072	P	aromatic amino acid family metabolic process	219	236	2.9E-10	1.7E-08	7.76955108
GO:0019395	P	fatty acid oxidation	165	174	3.4E-10	2E-08	7.69897
GO:0030258	P	lipid modification	204	219	4.4E-10	2.6E-08	7.58502665
GO:0000956	P	nuclear-transcribed mRNA catabolic process	107	109	4.7E-10	2.7E-08	7.56863624
GO:0006402	P	mRNA catabolic process	107	109	4.7E-10	2.7E-08	7.56863624
GO:0034440	P	lipid oxidation	170	180	5.2E-10	3E-08	7.52287875
GO:0071445	P	cellular response to protein stimulus	170	180	5.2E-10	3E-08	7.52287875
GO:0043574	P	peroxisomal transport	96	97	5.5E-10	3.2E-08	7.49485002
GO:0031048	P	chromatin silencing by small RNA	115	118	6.4E-10	3.7E-08	7.43179828
GO:0006625	P	protein targeting to peroxisome	95	96	7E-10	4E-08	7.39794001
GO:0030384	P	phosphoinositide metabolic process	147	154	7E-10	4E-08	7.39794001

GO:0016881	F	acid-amino acid ligase activity	267	291	1.1E-10	5.2E-08	7.28399666
GO:0006487	P	protein amino acid N-linked glycosylation	104	106	9.5E-10	5.4E-08	7.26760624
GO:0009640	P	photomorphogenesis	206	222	9.7E-10	5.5E-08	7.25963731
GO:0016558	P	protein import into peroxisome matrix	93	94	1.1E-09	6.4E-08	7.19382003
GO:0006661	P	phosphatidylinositol biosynthetic process	93	94	1.1E-09	6.4E-08	7.19382003
GO:0009067	P	aspartate family amino acid biosynthetic process	121	125	1.2E-09	6.6E-08	7.18045606
GO:0009737	P	response to abscisic acid stimulus	511	585	1.2E-09	7E-08	7.15490196
GO:0000375	P	RNA splicing, via transesterification reactions	112	115	1.3E-09	7.1E-08	7.14874165
GO:0000377	P	RNA splicing, via transesterification reactions with bulged adenosine as nucleophile	112	115	1.3E-09	7.1E-08	7.14874165
GO:0071216	P	cellular response to biotic stimulus	172	183	1.3E-09	7.4E-08	7.13076828
GO:0032555	F	purine ribonucleotide binding	1835	2207	1.8E-10	7.5E-08	7.12493874
GO:0032553	F	ribonucleotide binding	1835	2207	1.8E-10	7.5E-08	7.12493874
GO:0034976	P	response to endoplasmic reticulum stress	302	335	1.4E-09	7.7E-08	7.11350927
GO:0046165	P	alcohol biosynthetic process	257	282	1.5E-09	8.3E-08	7.08092191
GO:0000280	P	nuclear division	102	104	1.5E-09	8.4E-08	7.07572071
GO:0009743	P	response to carbohydrate stimulus	668	776	1.7E-09	9.3E-08	7.03151705
GO:0007126	P	meiosis	190	204	1.8E-09	9.8E-08	7.00877392
GO:0051327	P	M phase of meiotic cell cycle	190	204	1.8E-09	9.8E-08	7.00877392
GO:0034504	P	protein localization in nucleus	142	149	2E-09	1.1E-07	6.95860731
GO:0006986	P	response to unfolded protein	163	173	2E-09	1.1E-07	6.95860731

GO:0034620	P	cellular response to unfolded protein	163	173	2E-09	1.1E-07	6.95860731
GO:0007059	P	chromosome segregation	149	157	2.1E-09	1.1E-07	6.95860731
GO:0016071	P	mRNA metabolic process	320	357	2.3E-09	1.3E-07	6.88605665
GO:0051170	P	nuclear import	141	148	2.4E-09	1.3E-07	6.88605665
GO:0005635	C	nuclear envelope	76	76	3.9E-09	1.3E-07	6.88605665
GO:0022627	C	cytosolic small ribosomal subunit	98	100	3.9E-09	1.3E-07	6.88605665
GO:0009579	C	thylakoid	413	469	4.2E-09	1.3E-07	6.88605665
GO:0006816	P	calcium ion transport	117	121	2.8E-09	1.5E-07	6.82390874
GO:0006984	P	ER-nuclear signaling pathway	168	179	2.9E-09	1.5E-07	6.82390874
GO:0010090	P	trichome morphogenesis	140	147	3E-09	1.6E-07	6.79588002
GO:0030968	P	endoplasmic reticulum unfolded protein response	161	171	3E-09	1.6E-07	6.79588002
GO:0009451	P	RNA modification	318	355	3.1E-09	1.7E-07	6.76955108
GO:0006606	P	protein import into nucleus	139	146	3.7E-09	1.9E-07	6.7212464
GO:0009933	P	meristem structural organization	222	242	4.1E-09	2.1E-07	6.67778071
GO:0044425	C	membrane part	1839	2226	6.7E-09	2.1E-07	6.67778071
GO:0009909	P	regulation of flower development	305	340	4.4E-09	2.3E-07	6.63827216
GO:0000302	P	response to reactive oxygen species	294	327	4.6E-09	2.4E-07	6.61978876
GO:0034284	P	response to monosaccharide stimulus	158	168	5.4E-09	2.8E-07	6.55284197
GO:0009746	P	response to hexose stimulus	158	168	5.4E-09	2.8E-07	6.55284197
GO:0032446	P	protein modification by small protein conjugation	237	260	6E-09	3.1E-07	6.50863831
GO:0048532	P	anatomical structure arrangement	225	246	6.5E-09	3.4E-07	6.46852108
GO:0009750	P	response to fructose stimulus	136	143	6.8E-09	3.5E-07	6.45593196
GO:0018130	P	heterocycle biosynthetic process	230	252	7.4E-09	3.8E-07	6.4202164
GO:0046488	P	phosphatidylinositol metabolic process	104	107	7.9E-09	4E-07	6.39794001
GO:0051325	P	interphase	73	73	8.4E-09	4.3E-07	6.36653154
GO:0008026	F	ATP-dependent helicase activity	112	115	1.3E-09	4.7E-07	6.32790214
GO:0070035	F	purine NTP-dependent helicase activity	112	115	1.3E-09	4.7E-07	6.32790214
GO:0051329	P	interphase of mitotic cell cycle	72	72	1.1E-08	5.5E-07	6.25963731
GO:0005524	F	ATP binding	1513	1816	1.8E-09	5.7E-07	6.24412514
GO:0019787	F	small conjugating protein ligase activity	239	261	1.7E-09	5.7E-07	6.24412514

GO:0044455	C	mitochondrial membrane part	131	138	1.9E-08	5.9E-07	6.22914799
GO:0004518	F	nuclease activity	156	165	2.1E-09	6.5E-07	6.18708664
GO:0070838	P	divalent metal ion transport	179	193	1.3E-08	6.7E-07	6.1739252
GO:0051168	P	nuclear export	71	71	1.4E-08	7E-07	6.15490196
GO:0050789	P	regulation of biological process	3777	4655	1.5E-08	7.5E-07	6.12493874
GO:0000398	P	nuclear mRNA splicing, via spliceosome	101	104	1.6E-08	7.8E-07	6.1079054
GO:0006163	P	purine nucleotide metabolic process	178	192	1.6E-08	8E-07	6.09691001
GO:0033013	P	tetrapyrrole metabolic process	208	227	1.6E-08	8E-07	6.09691001
GO:0006914	P	autophagy	70	70	1.8E-08	8.9E-07	6.05060999
GO:0000271	P	polysaccharide biosynthetic process	502	579	1.8E-08	9E-07	6.04575749
GO:0006778	P	porphyrin metabolic process	207	226	1.9E-08	9.4E-07	6.02687215
GO:0004842	F	ubiquitin-protein ligase activity	235	257	3.3E-09	9.6E-07	6.01772877
GO:0033554	P	cellular response to stress	1162	1390	2.3E-08	1.1E-06	5.95860731
GO:0033043	P	regulation of organelle organization	157	168	2.3E-08	1.1E-06	5.95860731
GO:0000086	P	G2/M transition of mitotic cell cycle	69	69	2.3E-08	1.1E-06	5.95860731
GO:0044430	C	cytoskeletal part	148	158	3.7E-08	1.1E-06	5.95860731
GO:0009963	P	positive regulation of flavonoid biosynthetic process	99	102	2.4E-08	1.2E-06	5.92081875
GO:0022610	P	biological adhesion	90	92	2.5E-08	1.2E-06	5.92081875
GO:0007155	P	cell adhesion	90	92	2.5E-08	1.2E-06	5.92081875
GO:0009561	P	megagametogenesis	169	182	2.6E-08	1.3E-06	5.88605665
GO:0007062	P	sister chromatid cohesion	129	136	2.8E-08	1.4E-06	5.85387196
GO:0010389	P	regulation of G2/M transition of mitotic cell cycle	68	68	3E-08	1.4E-06	5.85387196
GO:0048583	P	regulation of response to stimulus	557	647	3.2E-08	1.6E-06	5.79588002
GO:0008610	P	lipid biosynthetic process	915	1086	3.3E-08	1.6E-06	5.79588002
GO:0007034	P	vacuolar transport	121	127	3.3E-08	1.6E-06	5.79588002
GO:0050658	P	RNA transport	67	67	3.9E-08	1.8E-06	5.74472749
GO:0050657	P	nucleic acid transport	67	67	3.9E-08	1.8E-06	5.74472749
GO:0045010	P	actin nucleation	97	100	3.8E-08	1.8E-06	5.74472749
GO:0000087	P	M phase of mitotic cell cycle	67	67	3.9E-08	1.8E-06	5.74472749
GO:0051236	P	establishment of RNA localization	67	67	3.9E-08	1.8E-06	5.74472749

GO:0006403	P	RNA localization	67	67	3.9E-08	1.8E-06	5.74472749
GO:0006405	P	RNA export from nucleus	67	67	3.9E-08	1.8E-06	5.74472749
GO:0006766	P	vitamin metabolic process	141	150	4E-08	1.9E-06	5.7212464
GO:0070469	C	respiratory chain	86	88	6.4E-08	1.9E-06	5.7212464
GO:0016567	P	protein ubiquitination	219	241	4.4E-08	2.1E-06	5.67778071
GO:0010073	P	meristem maintenance	252	280	4.4E-08	2.1E-06	5.67778071
GO:0045036	P	protein targeting to chloroplast	66	66	5E-08	2.3E-06	5.63827216
GO:0007020	P	microtubule nucleation	66	66	5E-08	2.3E-06	5.63827216
GO:0006270	P	DNA replication initiation	66	66	5E-08	2.3E-06	5.63827216
GO:0007067	P	mitosis	66	66	5E-08	2.3E-06	5.63827216
GO:0044427	C	chromosomal part	131	139	7.6E-08	2.3E-06	5.63827216
GO:0006623	P	protein targeting to vacuole	111	116	5.7E-08	2.6E-06	5.58502665
GO:0070887	P	cellular response to chemical stimulus	1097	1313	6.6E-08	0.000003	5.52287875
GO:0009629	P	response to gravity	151	162	7.1E-08	3.2E-06	5.49485002
GO:0031984	C	organelle subcompartment	343	390	1.1E-07	3.2E-06	5.49485002
GO:0016144	P	S-glycoside biosynthetic process	157	169	7.5E-08	3.4E-06	5.46852108
GO:0019761	P	glucosinolate biosynthetic process	157	169	7.5E-08	3.4E-06	5.46852108
GO:0010075	P	regulation of meristem growth	157	169	7.5E-08	3.4E-06	5.46852108
GO:0019758	P	glycosinolate biosynthetic process	157	169	7.5E-08	3.4E-06	5.46852108
GO:0009108	P	coenzyme biosynthetic process	144	154	7.9E-08	3.6E-06	5.4436975
GO:0005746	C	mitochondrial respiratory chain	83	85	1.3E-07	3.8E-06	5.4202164
GO:0015630	C	microtubule cytoskeleton	114	120	1.4E-07	4.1E-06	5.38721614
GO:0006089	P	lactate metabolic process	93	96	9.4E-08	4.2E-06	5.37675071
GO:0015994	P	chlorophyll metabolic process	174	189	9.4E-08	4.2E-06	5.37675071
GO:0031976	C	plastid thylakoid	340	387	1.6E-07	4.4E-06	5.35654732
GO:0009534	C	chloroplast thylakoid	340	387	1.6E-07	4.4E-06	5.35654732
GO:0046700	P	heterocycle catabolic process	224	248	1.1E-07	5.1E-06	5.29242982
GO:0046185	P	aldehyde catabolic process	92	95	1.2E-07	5.2E-06	5.28399666
GO:0006457	P	protein folding	287	323	1.2E-07	5.3E-06	5.27572413
GO:0009606	P	tropism	154	166	1.3E-07	5.8E-06	5.23657201

GO:0006084	P	acetyl-CoA metabolic process	107	112	1.3E-07	5.9E-06	5.22914799
GO:0051640	P	organelle localization	141	151	1.4E-07	6.2E-06	5.20760831
GO:0005976	P	polysaccharide metabolic process	592	693	1.4E-07	6.3E-06	5.20065945
GO:0010014	P	meristem initiation	153	165	1.6E-07	6.8E-06	5.16749109
GO:0042542	P	response to hydrogen peroxide	227	252	1.7E-07	7.3E-06	5.13667714
GO:0005777	C	peroxisome	162	176	2.7E-07	7.4E-06	5.13076828
GO:0042579	C	microbody	162	176	2.7E-07	7.4E-06	5.13076828
GO:0016572	P	histone phosphorylation	61	61	1.8E-07	7.8E-06	5.1079054
GO:0009438	P	methylglyoxal metabolic process	90	93	1.8E-07	0.000008	5.09691001
GO:0051596	P	methylglyoxal catabolic process	90	93	1.8E-07	0.000008	5.09691001
GO:0051321	P	meiotic cell cycle	226	251	1.9E-07	8.4E-06	5.07572071
GO:0009310	P	amine catabolic process	187	205	2E-07	8.7E-06	5.06048075
GO:0061024	P	membrane organization	456	528	2.2E-07	9.5E-06	5.02227639
GO:0016044	P	cellular membrane organization	456	528	2.2E-07	9.5E-06	5.02227639
GO:0016126	P	sterol biosynthetic process	151	163	2.2E-07	9.6E-06	5.01772877
GO:0016125	P	sterol metabolic process	157	170	2.3E-07	9.7E-06	5.01322827
GO:0019243	P	methylglyoxal catabolic process to D-lactate	89	92	2.3E-07	9.7E-06	5.01322827
GO:0010212	P	response to ionizing radiation	89	92	2.3E-07	9.7E-06	5.01322827
GO:0006406	P	mRNA export from nucleus	60	60	2.3E-07	9.7E-06	5.01322827
GO:0051028	P	mRNA transport	60	60	2.3E-07	9.7E-06	5.01322827
GO:0042793	P	transcription from plastid promoter	71	72	2.4E-07	0.00001	5
GO:0006302	P	double-strand break repair	104	109	2.5E-07	0.000011	4.95860731
GO:0035266	P	meristem growth	162	176	2.7E-07	0.000011	4.95860731
GO:0009553	P	embryo sac development	213	236	2.7E-07	0.000011	4.95860731
GO:0042744	P	hydrogen peroxide catabolic process	70	71	3E-07	0.000013	4.88605665
GO:0009630	P	gravitropism	143	154	3.1E-07	0.000013	4.88605665
GO:0046417	P	chorismate metabolic process	95	99	3.5E-07	0.000014	4.85387196
GO:0034285	P	response to disaccharide stimulus	189	208	3.6E-07	0.000015	4.82390874
GO:0007015	P	actin filament organization	142	153	3.7E-07	0.000015	4.82390874
GO:0016926	P	protein desumoylation	78	80	4.1E-07	0.000017	4.76955108

GO:0006631	P	fatty acid metabolic process	482	561	4.2E-07	0.000017	4.76955108
GO:0045271	C	respiratory chain complex I	56	56	6.3E-07	0.000017	4.76955108
GO:0000502	C	proteasome complex	56	56	6.3E-07	0.000017	4.76955108
GO:0030964	C	NADH dehydrogenase complex	56	56	6.3E-07	0.000017	4.76955108
GO:0009073	P	aromatic amino acid family biosynthetic process	94	98	4.3E-07	0.000018	4.74472749
GO:0051604	P	protein maturation	77	79	5.1E-07	0.000021	4.67778071
GO:0006833	P	water transport	140	151	5.3E-07	0.000022	4.65757732
GO:0042044	P	fluid transport	140	151	5.3E-07	0.000022	4.65757732
GO:0006767	P	water-soluble vitamin metabolic process	93	97	5.3E-07	0.000022	4.65757732
GO:0009744	P	response to sucrose stimulus	186	205	5.9E-07	0.000024	4.61978876
GO:0010050	P	vegetative phase change	67	68	6.2E-07	0.000025	4.60205999
GO:0007623	P	circadian rhythm	151	164	6.5E-07	0.000026	4.58502665
GO:0048511	P	rhythmic process	151	164	6.5E-07	0.000026	4.58502665
GO:0040008	P	regulation of growth	259	292	6.8E-07	0.000028	4.55284197
GO:0051667	P	establishment of plastid localization	99	104	7.1E-07	0.000028	4.55284197
GO:0009902	P	chloroplast relocation	99	104	7.1E-07	0.000028	4.55284197
GO:0006888	P	ER to Golgi vesicle-mediated transport	99	104	7.1E-07	0.000028	4.55284197
GO:0051644	P	plastid localization	99	104	7.1E-07	0.000028	4.55284197
GO:0005747	C	mitochondrial respiratory chain complex I	54	54	1.1E-06	0.000028	4.55284197
GO:0048507	P	meristem development	412	477	7.7E-07	0.000031	4.50863831
GO:0005856	C	cytoskeleton	175	193	1.4E-06	0.000036	4.4436975
GO:0016042	P	lipid catabolic process	226	253	9.2E-07	0.000037	4.43179828
GO:0009062	P	fatty acid catabolic process	194	215	9.4E-07	0.000037	4.43179828
GO:0016779	F	nucleotidyltransferase activity	154	166	1.3E-07	0.000037	4.43179828
GO:0016853	F	isomerase activity	200	220	1.5E-07	0.00004	4.39794001
GO:0017076	F	purine nucleotide binding	1933	2357	1.5E-07	0.00004	4.39794001
GO:0032559	F	adenyl ribonucleotide binding	1626	1974	1.6E-07	0.00004	4.39794001
GO:0016579	P	protein deubiquitination	54	54	1.1E-06	0.000042	4.37675071
GO:0016138	P	glycoside biosynthetic process	187	207	1.2E-06	0.000047	4.32790214
GO:0032504	P	multicellular organism reproduction	328	376	1.2E-06	0.000049	4.30980392

GO:0023034	P	intracellular signaling pathway	347	399	1.2E-06	0.000049	4.30980392
GO:0033014	P	tetrapyrrole biosynthetic process	147	160	1.3E-06	0.000051	4.29242982
GO:0044436	C	thylakoid part	305	349	0.000002	0.000051	4.29242982
GO:0023033	P	signaling pathway	411	477	1.4E-06	0.000056	4.25181197
GO:0034357	C	photosynthetic membrane	275	313	2.2E-06	0.000056	4.25181197
GO:0002376	P	immune system process	798	953	1.6E-06	0.000062	4.20760831
GO:0006955	P	immune response	798	953	1.6E-06	0.000062	4.20760831
GO:0044242	P	cellular lipid catabolic process	217	243	1.6E-06	0.000062	4.20760831
GO:0048825	P	cotyledon development	63	64	1.6E-06	0.000063	4.20065945
GO:0042651	C	thylakoid membrane	269	306	2.5E-06	0.000064	4.19382003
GO:0033044	P	regulation of chromosome organization	115	123	1.7E-06	0.000065	4.18708664
GO:0009642	P	response to light intensity	242	273	1.7E-06	0.000067	4.1739252
GO:0071310	P	cellular response to organic substance	954	1147	1.9E-06	0.000074	4.13076828
GO:0051656	P	establishment of organelle localization	101	107	1.9E-06	0.000075	4.12493874
GO:0010557	P	positive regulation of macromolecule biosynthetic process	394	457	0.000002	0.000077	4.11350927
GO:0051130	P	positive regulation of cellular component organization	62	63	2.1E-06	0.000079	4.10237291
GO:0006779	P	porphyrin biosynthetic process	144	157	2.2E-06	0.000083	4.08092191
GO:0010604	P	positive regulation of macromolecule metabolic process	406	472	2.4E-06	0.000092	4.03621217
GO:0042558	P	pteridine and derivative metabolic process	61	62	2.6E-06	0.000099	4.00436481
GO:0006164	P	purine nucleotide biosynthetic process	99	105	2.9E-06	0.00011	3.95860731
GO:0042170	C	plastid membrane	90	95	4.5E-06	0.00011	3.95860731
GO:0080134	P	regulation of response to stress	453	530	3.2E-06	0.00012	3.92081875
GO:0050665	P	hydrogen peroxide biosynthetic process	77	80	3.2E-06	0.00012	3.92081875
GO:0055035	C	plastid thylakoid membrane	254	289	4.9E-06	0.00012	3.92081875
GO:0048609	P	reproductive process in a multicellular organism	305	350	3.5E-06	0.00013	3.88605665
GO:0019001	F	guanyl nucleotide binding	219	244	5.5E-07	0.00013	3.88605665
GO:0007276	P	gamete generation	169	187	3.6E-06	0.00014	3.85387196
GO:0009250	P	glucan biosynthetic process	271	309	3.7E-06	0.00014	3.85387196
GO:0009150	P	purine ribonucleotide metabolic process	146	160	4.1E-06	0.00015	3.82390874
GO:0009535	C	chloroplast thylakoid membrane	252	287	6.4E-06	0.00016	3.79588002

GO:0031347	P	regulation of defense response	440	515	4.7E-06	0.00018	3.74472749
GO:0015672	P	monovalent inorganic cation transport	199	223	4.7E-06	0.00018	3.74472749
GO:0007051	P	spindle organization	48	48	4.8E-06	0.00018	3.74472749
GO:0007018	P	microtubule-based movement	67	69	0.000005	0.00019	3.7212464
GO:0009409	P	response to cold	501	590	5.1E-06	0.00019	3.7212464
GO:0005525	F	GTP binding	216	241	8.6E-07	0.00019	3.7212464
GO:0032561	F	guanyl ribonucleotide binding	216	241	8.6E-07	0.00019	3.7212464
GO:0044042	P	glucan metabolic process	362	420	5.3E-06	0.0002	3.69897
GO:0042175	C	nuclear envelope-endoplasmic reticulum network	87	92	8.2E-06	0.0002	3.69897
GO:0016887	F	ATPase activity	396	458	9.8E-07	0.00021	3.67778071
GO:0010026	P	trichome differentiation	149	164	6.3E-06	0.00023	3.63827216
GO:0035315	P	hair cell differentiation	149	164	6.3E-06	0.00023	3.63827216
GO:0000725	P	recombinational repair	66	68	6.3E-06	0.00023	3.63827216
GO:0000724	P	double-strand break repair via homologous recombination	66	68	6.3E-06	0.00023	3.63827216
GO:0048827	P	phyllome development	401	468	6.5E-06	0.00024	3.61978876
GO:0016791	F	phosphatase activity	245	276	1.2E-06	0.00024	3.61978876
GO:0010638	P	positive regulation of organelle organization	57	58	6.8E-06	0.00025	3.60205999
GO:0042578	F	phosphoric ester hydrolase activity	304	347	1.2E-06	0.00025	3.60205999
GO:0071322	P	cellular response to carbohydrate stimulus	107	115	7.5E-06	0.00027	3.56863624
GO:0015992	P	proton transport	119	129	7.7E-06	0.00028	3.55284197
GO:0016143	P	S-glycoside metabolic process	180	201	7.7E-06	0.00028	3.55284197
GO:0006818	P	hydrogen transport	119	129	7.7E-06	0.00028	3.55284197
GO:0019760	P	glucosinolate metabolic process	180	201	7.7E-06	0.00028	3.55284197
GO:0006612	P	protein targeting to membrane	331	383	7.7E-06	0.00028	3.55284197
GO:0019757	P	glycosinolate metabolic process	180	201	7.7E-06	0.00028	3.55284197
GO:0070925	P	organelle assembly	56	57	8.6E-06	0.00031	3.50863831
GO:0048638	P	regulation of developmental growth	230	261	8.6E-06	0.00031	3.50863831
GO:0051128	P	regulation of cellular component organization	230	261	8.6E-06	0.00031	3.50863831
GO:0006629	P	lipid metabolic process	1353	1651	9.4E-06	0.00034	3.46852108
GO:0048509	P	regulation of meristem development	194	218	9.7E-06	0.00035	3.45593196

GO:0051173	P	positive regulation of nitrogen compound metabolic process	383	447	0.00001	0.00036	3.4436975
GO:0005789	C	endoplasmic reticulum membrane	84	89	0.000015	0.00036	3.4436975
GO:0051225	P	spindle assembly	45	45	0.00001	0.00037	3.43179828
GO:0030036	P	actin cytoskeleton organization	162	180	0.000011	0.00038	3.4202164
GO:0033692	P	cellular polysaccharide biosynthetic process	436	512	0.000011	0.00038	3.4202164
GO:0045132	P	meiotic chromosome segregation	105	113	0.000011	0.00038	3.4202164
GO:0010033	P	response to organic substance	2095	2583	0.000011	0.00038	3.4202164
GO:0045935	P	positive regulation of nucleobase, nucleoside, nucleotide and nucleic acid metabolic process	382	446	0.000011	0.0004	3.39794001
GO:0016311	P	dephosphorylation	213	241	0.000011	0.0004	3.39794001
GO:0003676	F	nucleic acid binding	2402	2959	2.1E-06	0.0004	3.39794001
GO:0010332	P	response to gamma radiation	71	74	0.000012	0.00041	3.38721614
GO:0070301	P	cellular response to hydrogen peroxide	71	74	0.000012	0.00041	3.38721614
GO:0009063	P	cellular amino acid catabolic process	155	172	0.000014	0.00048	3.31875876
GO:0007033	P	vacuole organization	54	55	0.000014	0.00048	3.31875876
GO:0043648	P	dicarboxylic acid metabolic process	126	138	0.000017	0.0006	3.22184875
GO:0009880	P	embryonic pattern specification	43	43	0.000017	0.0006	3.22184875
GO:0003924	F	GTPase activity	105	112	3.3E-06	0.00061	3.21467016
GO:0071212	C	subsynaptic reticulum	94	101	0.000026	0.00061	3.21467016
GO:0044432	C	endoplasmic reticulum part	94	101	0.000026	0.00061	3.21467016
GO:0031969	C	chloroplast membrane	81	86	0.000027	0.00063	3.20065945
GO:0006354	P	RNA elongation	61	63	0.00002	0.00068	3.16749109
GO:0009408	P	response to heat	252	289	0.000021	0.00073	3.13667714
GO:0045941	P	positive regulation of transcription	371	434	0.000022	0.00075	3.12493874
GO:0045893	P	positive regulation of transcription, DNA-dependent	371	434	0.000022	0.00075	3.12493874
GO:0042402	P	cellular biogenic amine catabolic process	101	109	0.000022	0.00077	3.11350927
GO:0044454	C	nuclear chromosome part	40	40	0.000037	0.00085	3.07058107
GO:0051254	P	positive regulation of RNA metabolic process	374	438	0.000025	0.00087	3.06048075
GO:0010628	P	positive regulation of gene expression	374	438	0.000025	0.00087	3.06048075
GO:0008094	F	DNA-dependent ATPase activity	48	48	4.8E-06	0.00088	3.05551733
GO:0009855	P	determination of bilateral symmetry	112	122	0.000026	0.00089	3.05060999

GO:0016226	P	iron-sulfur cluster assembly	94	101	0.000026	0.00089	3.05060999
GO:0031163	P	metallo-sulfur cluster assembly	94	101	0.000026	0.00089	3.05060999
GO:0010207	P	photosystem II assembly	140	155	0.000026	0.00089	3.05060999
GO:0034050	P	host programmed cell death induced by symbiont	337	393	0.000028	0.00097	3.01322827
GO:0006366	P	transcription from RNA polymerase II promoter	74	78	0.000029	0.00097	3.01322827
GO:0044093	P	positive regulation of molecular function	117	128	0.00003	0.001	3
GO:0030135	C	coated vesicle	49	50	0.000045	0.001	3
GO:0009528	C	plastid inner membrane	49	50	0.000045	0.001	3
GO:0009626	P	plant-type hypersensitive response	336	392	0.000032	0.0011	2.95860731
GO:0015979	P	photosynthesis	304	353	0.000033	0.0011	2.95860731
GO:0045087	P	innate immune response	748	901	0.000033	0.0011	2.95860731
GO:0043067	P	regulation of programmed cell death	331	386	0.000033	0.0011	2.95860731
GO:0004721	F	phosphoprotein phosphatase activity	160	177	6.3E-06	0.0011	2.95860731
GO:0043085	P	positive regulation of catalytic activity	116	127	0.000035	0.0012	2.92081875
GO:0043038	P	amino acid activation	50	51	0.000036	0.0012	2.92081875
GO:0043039	P	tRNA aminoacylation	50	51	0.000036	0.0012	2.92081875
GO:0006418	P	tRNA aminoacylation for protein translation	50	51	0.000036	0.0012	2.92081875
GO:0031988	C	membrane-bounded vesicle	64	67	0.000053	0.0012	2.92081875
GO:0016109	P	tetraterpenoid biosynthetic process	98	106	0.000039	0.0013	2.88605665
GO:0016117	P	carotenoid biosynthetic process	98	106	0.000039	0.0013	2.88605665
GO:0007127	P	meiosis I	126	139	0.000043	0.0014	2.85387196
GO:0012501	P	programmed cell death	373	438	0.000043	0.0014	2.85387196
GO:0030243	P	cellulose metabolic process	131	145	0.000046	0.0015	2.82390874
GO:0009825	P	multidimensional cell growth	97	105	0.000046	0.0015	2.82390874
GO:0007131	P	reciprocal meiotic recombination	120	132	0.000046	0.0015	2.82390874
GO:0051707	P	response to other organism	1109	1353	0.000047	0.0015	2.82390874
GO:0042440	P	pigment metabolic process	305	355	0.000047	0.0016	2.79588002
GO:0016137	P	glycoside metabolic process	226	259	0.000049	0.0016	2.79588002
GO:0015995	P	chlorophyll biosynthetic process	114	125	0.000049	0.0016	2.79588002
GO:0010941	P	regulation of cell death	336	393	0.00005	0.0016	2.79588002

GO:0080135	P	regulation of cellular response to stress	318	371	0.00005	0.0016	2.79588002
GO:0004540	F	ribonuclease activity	72	75	9.5E-06	0.0016	2.79588002
GO:0071215	P	cellular response to abscisic acid stimulus	216	247	0.000051	0.0017	2.76955108
GO:0006972	P	hyperosmotic response	216	247	0.000051	0.0017	2.76955108
GO:0006333	P	chromatin assembly or disassembly	108	118	0.000052	0.0017	2.76955108
GO:0009110	P	vitamin biosynthetic process	71	75	0.000053	0.0017	2.76955108
GO:0048439	P	flower morphogenesis	64	67	0.000053	0.0017	2.76955108
GO:0009756	P	carbohydrate mediated signaling	96	104	0.000055	0.0018	2.74472749
GO:0010182	P	sugar mediated signaling pathway	96	104	0.000055	0.0018	2.74472749
GO:0048366	P	leaf development	370	435	0.000057	0.0018	2.74472749
GO:0051258	P	protein polymerization	56	58	0.00006	0.0019	2.7212464
GO:0010286	P	heat acclimation	77	82	0.00006	0.0019	2.7212464
GO:0006793	P	phosphorus metabolic process	1167	1427	0.000063	0.002	2.69897
GO:0016108	P	tetraterpenoid metabolic process	101	110	0.000064	0.002	2.69897
GO:0016116	P	carotenoid metabolic process	101	110	0.000064	0.002	2.69897
GO:0006752	P	group transfer coenzyme metabolic process	70	74	0.000064	0.002	2.69897
GO:0009706	C	chloroplast inner membrane	46	47	0.000091	0.002	2.69897
GO:0016604	C	nuclear body	46	47	0.000091	0.002	2.69897
GO:0009524	C	phragmoplast	46	47	0.000091	0.002	2.69897
GO:0010363	P	regulation of plant-type hypersensitive response	311	363	0.000065	0.0021	2.67778071
GO:0034599	P	cellular response to oxidative stress	89	96	0.000066	0.0021	2.67778071
GO:0006796	P	phosphate metabolic process	1166	1426	0.000067	0.0021	2.67778071
GO:0009799	P	specification of symmetry	112	123	0.000069	0.0022	2.65757732
GO:0019220	P	regulation of phosphate metabolic process	199	227	0.000071	0.0022	2.65757732
GO:0051174	P	regulation of phosphorus metabolic process	199	227	0.000071	0.0022	2.65757732
GO:0008135	F	translation factor activity, nucleic acid binding	116	126	0.000013	0.0022	2.65757732
GO:0070192	P	chromosome organization involved in meiosis	76	81	0.000073	0.0023	2.63827216
GO:0007129	P	synapsis	76	81	0.000073	0.0023	2.63827216
GO:0006414	P	translational elongation	47	48	0.000072	0.0023	2.63827216
GO:0009966	P	regulation of signal transduction	149	167	0.000073	0.0023	2.63827216

GO:0009141	P	nucleoside triphosphate metabolic process	128	142	0.000073	0.0023	2.63827216
GO:0023051	P	regulation of signaling process	149	167	0.000073	0.0023	2.63827216
GO:0002252	P	immune effector process	232	267	0.000074	0.0023	2.63827216
GO:0007242	P	intracellular signaling cascade	955	1163	0.000087	0.0027	2.56863624
GO:0051234	P	establishment of localization	2686	3343	0.00009	0.0028	2.55284197
GO:0006312	P	mitotic recombination	54	56	0.000093	0.0029	2.537602
GO:0034614	P	cellular response to reactive oxygen species	81	87	0.000093	0.0029	2.537602
GO:0016592	C	mediator complex	35	35	0.00013	0.0029	2.537602
GO:0050790	P	regulation of catalytic activity	262	304	0.000097	0.003	2.52287875
GO:0010646	P	regulation of cell communication	152	171	0.000099	0.003	2.52287875
GO:0046471	P	phosphatidylglycerol metabolic process	61	64	0.000099	0.003	2.52287875
GO:0009144	P	purine nucleoside triphosphate metabolic process	126	140	0.000099	0.003	2.52287875
GO:0009205	P	purine ribonucleoside triphosphate metabolic process	126	140	0.000099	0.003	2.52287875
GO:0009199	P	ribonucleoside triphosphate metabolic process	126	140	0.000099	0.003	2.52287875
GO:0008047	F	enzyme activator activity	76	80	0.000019	0.0031	2.50863831
GO:0008287	C	protein serine/threonine phosphatase complex	52	54	0.00014	0.0031	2.50863831
GO:0006733	P	oxidoreduction coenzyme metabolic process	229	264	0.0001	0.0032	2.49485002
GO:0008219	P	cell death	411	487	0.00011	0.0032	2.49485002
GO:0016265	P	death	411	487	0.00011	0.0032	2.49485002
GO:0009411	P	response to UV	209	240	0.00012	0.0036	2.4436975
GO:0046777	P	protein amino acid autophosphorylation	130	145	0.00012	0.0036	2.4436975
GO:0019207	F	kinase regulator activity	42	42	0.000022	0.0036	2.4436975
GO:0006655	P	phosphatidylglycerol biosynthetic process	60	63	0.00012	0.0037	2.43179828
GO:0051179	P	localization	2805	3496	0.00012	0.0037	2.43179828
GO:0000228	C	nuclear chromosome	51	53	0.00018	0.0038	2.4202164
GO:0031410	C	cytoplasmic vesicle	58	61	0.00019	0.0039	2.40893539
GO:0016023	C	cytoplasmic membrane-bounded vesicle	58	61	0.00019	0.0039	2.40893539
GO:0006301	P	postreplication repair	35	35	0.00013	0.004	2.39794001
GO:0042732	P	D-xylose metabolic process	35	35	0.00013	0.004	2.39794001
GO:0071103	P	DNA conformation change	91	99	0.00013	0.004	2.39794001

GO:0009749	P	response to glucose stimulus	79	85	0.00014	0.004	2.39794001
GO:0006470	P	protein amino acid dephosphorylation	184	210	0.00014	0.0041	2.38721614
GO:0019899	F	enzyme binding	134	148	0.000029	0.0043	2.36653154
GO:0015631	F	tubulin binding	51	52	0.000028	0.0043	2.36653154
GO:0006576	P	cellular biogenic amine metabolic process	149	168	0.00015	0.0045	2.34678749
GO:0009612	P	response to mechanical stimulus	59	62	0.00015	0.0045	2.34678749
GO:0060249	P	anatomical structure homeostasis	59	62	0.00015	0.0045	2.34678749
GO:0009644	P	response to high light intensity	188	215	0.00015	0.0046	2.33724217
GO:0001882	F	nucleoside binding	1729	2129	0.000032	0.0047	2.32790214
GO:0042325	P	regulation of phosphorylation	84	91	0.00016	0.0048	2.31875876
GO:0005982	P	starch metabolic process	197	226	0.00016	0.0048	2.31875876
GO:0005819	C	spindle	42	43	0.00023	0.0048	2.31875876
GO:0043603	P	cellular amide metabolic process	192	220	0.00017	0.005	2.30103
GO:0005488	F	binding	7321	9207	0.000036	0.005	2.30103
GO:0008092	F	cytoskeletal protein binding	127	140	0.000036	0.005	2.30103
GO:0008237	F	metallopeptidase activity	73	77	0.000035	0.005	2.30103
GO:0019887	F	protein kinase regulator activity	40	40	0.000037	0.005	2.30103
GO:0009755	P	hormone-mediated signaling pathway	456	544	0.00017	0.0051	2.29242982
GO:0009414	P	response to water deprivation	345	407	0.00017	0.0051	2.29242982
GO:0051540	F	metal cluster binding	72	76	0.000043	0.0052	2.28399666
GO:0043566	F	structure-specific DNA binding	65	68	0.000043	0.0052	2.28399666
GO:0001883	F	purine nucleoside binding	1722	2122	0.000043	0.0052	2.28399666
GO:0003774	F	motor activity	79	84	0.00004	0.0052	2.28399666
GO:0051536	F	iron-sulfur cluster binding	72	76	0.000043	0.0052	2.28399666
GO:0016875	F	ligase activity, forming carbon-oxygen bonds	49	50	0.000045	0.0052	2.28399666
GO:0016876	F	ligase activity, forming aminoacyl-tRNA and related compounds	49	50	0.000045	0.0052	2.28399666
GO:0004812	F	aminoacyl-tRNA ligase activity	49	50	0.000045	0.0052	2.28399666
GO:0030554	F	adenyl nucleotide binding	1720	2120	0.000047	0.0053	2.27572413
GO:0009611	P	response to wounding	283	331	0.00018	0.0054	2.26760624
GO:0042752	P	regulation of circadian rhythm	43	44	0.00018	0.0054	2.26760624

GO:0032200	P	telomere organization	58	61	0.00019	0.0054	2.26760624
GO:0000723	P	telomere maintenance	58	61	0.00019	0.0054	2.26760624
GO:0006073	P	cellular glucan metabolic process	322	379	0.0002	0.0057	2.24412514
GO:0042592	P	homeostatic process	390	463	0.00021	0.0062	2.20760831
GO:0009738	P	abscisic acid mediated signaling pathway	204	235	0.00022	0.0063	2.20065945
GO:0016197	P	endosome transport	33	33	0.00022	0.0064	2.19382003
GO:0042364	P	water-soluble vitamin biosynthetic process	57	60	0.00023	0.0066	2.18045606
GO:0006760	P	folic acid and derivative metabolic process	57	60	0.00023	0.0066	2.18045606
GO:0009820	P	alkaloid metabolic process	194	223	0.00024	0.0068	2.16749109
GO:0051246	P	regulation of protein metabolic process	194	223	0.00024	0.0068	2.16749109
GO:0009845	P	seed germination	189	217	0.00024	0.007	2.15490196
GO:0012506	C	vesicle membrane	40	41	0.00037	0.0074	2.13076828
GO:0031980	C	mitochondrial lumen	40	41	0.00037	0.0074	2.13076828
GO:0005759	C	mitochondrial matrix	40	41	0.00037	0.0074	2.13076828
GO:0010051	P	xylem and phloem pattern formation	87	95	0.00027	0.0076	2.11918641
GO:0000785	C	chromatin	79	86	0.00039	0.0078	2.1079054
GO:0019684	P	photosynthesis, light reaction	243	283	0.00028	0.0081	2.09151498
GO:0045786	P	negative regulation of cell cycle	32	32	0.00029	0.0081	2.09151498
GO:0009081	P	branched chain family amino acid metabolic process	32	32	0.00029	0.0081	2.09151498
GO:0006469	P	negative regulation of protein kinase activity	41	42	0.00029	0.0084	2.07572071
GO:0051348	P	negative regulation of transferase activity	41	42	0.00029	0.0084	2.07572071
GO:0033673	P	negative regulation of kinase activity	41	42	0.00029	0.0084	2.07572071
GO:0000139	C	Golgi membrane	47	49	0.00043	0.0084	2.07572071
GO:0003777	F	microtubule motor activity	62	65	0.000081	0.0089	2.05060999
GO:0006891	P	intra-Golgi vesicle-mediated transport	62	66	0.00032	0.009	2.04575749
GO:0044433	C	cytoplasmic vesicle part	39	40	0.00047	0.0091	2.04095861
GO:0030659	C	cytoplasmic vesicle membrane	39	40	0.00047	0.0091	2.04095861
GO:0019867	C	outer membrane	72	78	0.00049	0.0093	2.03151705
GO:0032268	P	regulation of cellular protein metabolic process	181	208	0.00036	0.01	2
GO:0044264	P	cellular polysaccharide metabolic process	487	585	0.0004	0.011	1.95860731

GO:0019362	P	pyridine nucleotide metabolic process	184	212	0.00044	0.013	1.88605665
GO:0019252	P	starch biosynthetic process	165	189	0.00045	0.013	1.88605665
GO:0045859	P	regulation of protein kinase activity	78	85	0.00047	0.013	1.88605665
GO:0050826	P	response to freezing	78	85	0.00047	0.013	1.88605665
GO:0043549	P	regulation of kinase activity	78	85	0.00047	0.013	1.88605665
GO:0006417	P	regulation of translation	60	64	0.00047	0.013	1.88605665
GO:0019222	P	regulation of metabolic process	2217	2763	0.00047	0.013	1.88605665
GO:0008270	F	zinc ion binding	1041	1272	0.00012	0.013	1.88605665
GO:0031072	F	heat shock protein binding	114	126	0.00012	0.013	1.88605665
GO:0031968	C	organelle outer membrane	70	76	0.00069	0.013	1.88605665
GO:0030662	C	coated vesicle membrane	37	38	0.00074	0.014	1.85387196
GO:0032870	P	cellular response to hormone stimulus	483	581	0.00053	0.015	1.82390874
GO:0051082	F	unfolded protein binding	102	112	0.00014	0.015	1.82390874
GO:0005643	C	nuclear pore	28	28	0.00079	0.015	1.82390874
GO:0046930	C	pore complex	28	28	0.00079	0.015	1.82390874
GO:0006997	P	nucleus organization	77	84	0.00056	0.016	1.79588002
GO:0000737	P	DNA catabolic process, endonucleolytic	77	84	0.00056	0.016	1.79588002
GO:0046496	P	nicotinamide nucleotide metabolic process	182	210	0.00056	0.016	1.79588002
GO:0006769	P	nicotinamide metabolic process	182	210	0.00056	0.016	1.79588002
GO:0019321	P	pentose metabolic process	65	70	0.00059	0.016	1.79588002
GO:0031300	C	intrinsic to organelle membrane	63	68	0.00085	0.016	1.79588002
GO:0007264	P	small GTPase mediated signal transduction	124	140	0.0006	0.017	1.76955108
GO:0009850	P	auxin metabolic process	119	134	0.0006	0.017	1.76955108
GO:0004722	F	protein serine/threonine phosphatase activity	128	143	0.00016	0.017	1.76955108
GO:0005099	F	Ras GTPase activator activity	34	34	0.00017	0.017	1.76955108
GO:0030120	C	vesicle coat	36	37	0.00094	0.017	1.76955108
GO:0031301	C	integral to organelle membrane	50	53	0.00095	0.017	1.76955108
GO:0008033	P	tRNA processing	52	55	0.00064	0.018	1.74472749
GO:0050776	P	regulation of immune response	343	408	0.00065	0.018	1.74472749
GO:0002682	P	regulation of immune system process	343	408	0.00065	0.018	1.74472749

GO:0009066	P	aspartate family amino acid metabolic process	226	264	0.00066	0.018	1.74472749
GO:0042138	P	meiotic DNA double-strand break formation	76	83	0.00066	0.018	1.74472749
GO:0009415	P	response to water	347	413	0.00067	0.018	1.74472749
GO:0046658	C	anchored to plasma membrane	62	67	0.001	0.018	1.74472749
GO:0010008	C	endosome membrane	27	27	0.001	0.018	1.74472749
GO:0005874	C	microtubule	56	60	0.001	0.018	1.74472749
GO:0044440	C	endosomal part	27	27	0.001	0.018	1.74472749
GO:0009152	P	purine ribonucleotide biosynthetic process	70	76	0.00069	0.019	1.7212464
GO:0006810	P	transport	2613	3268	0.0007	0.019	1.7212464
GO:0018193	P	peptidyl-amino acid modification	475	572	0.00071	0.019	1.7212464
GO:0051338	P	regulation of transferase activity	81	89	0.00073	0.02	1.69897
GO:0046655	P	folic acid metabolic process	37	38	0.00074	0.02	1.69897
GO:0046482	P	para-aminobenzoic acid metabolic process	37	38	0.00074	0.02	1.69897
GO:0046039	P	GTP metabolic process	44	46	0.00082	0.022	1.65757732
GO:0008017	F	microtubule binding	42	43	0.00023	0.023	1.63827216
GO:0065007	P	biological regulation	4434	5583	0.00089	0.024	1.61978876
GO:0045088	P	regulation of innate immune response	339	404	0.00092	0.025	1.60205999
GO:0009805	P	coumarin biosynthetic process	50	53	0.00095	0.026	1.58502665
GO:0032204	P	regulation of telomere maintenance	50	53	0.00095	0.026	1.58502665
GO:0032844	P	regulation of homeostatic process	50	53	0.00095	0.026	1.58502665
GO:0006284	P	base-excision repair	27	27	0.001	0.027	1.56863624
GO:0045736	P	negative regulation of cyclin-dependent protein kinase activity	27	27	0.001	0.027	1.56863624
GO:0006665	P	sphingolipid metabolic process	62	67	0.001	0.027	1.56863624
GO:0009832	P	plant-type cell wall biogenesis	125	142	0.001	0.027	1.56863624
GO:0042221	P	response to chemical stimulus	2979	3736	0.001	0.027	1.56863624
GO:0016538	F	cyclin-dependent protein kinase regulator activity	32	32	0.00029	0.028	1.55284197
GO:0010431	P	seed maturation	139	159	0.0011	0.029	1.537602
GO:0008276	F	protein methyltransferase activity	41	42	0.00029	0.029	1.537602
GO:0005838	C	proteasome regulatory particle	25	25	0.0017	0.029	1.537602
GO:0005839	C	proteasome core complex	25	25	0.0017	0.029	1.537602

GO:0005798	C	Golgi-associated vesicle	25	25	0.0017	0.029	1.537602
GO:0022624	C	proteasome accessory complex	25	25	0.0017	0.029	1.537602
GO:0031497	P	chromatin assembly	67	73	0.0012	0.031	1.50863831
GO:0042623	F	ATPase activity, coupled	290	341	0.00034	0.032	1.49485002
GO:0048508	P	embryonic meristem development	55	59	0.0012	0.033	1.48148606
GO:0006739	P	NADP metabolic process	175	203	0.0012	0.033	1.48148606
GO:0000305	P	response to oxygen radical	42	44	0.0013	0.033	1.48148606
GO:0000303	P	response to superoxide	42	44	0.0013	0.033	1.48148606
GO:0006308	P	DNA catabolic process	83	92	0.0013	0.034	1.46852108
GO:0009410	P	response to xenobiotic stimulus	72	79	0.0013	0.034	1.46852108
GO:0003684	F	damaged DNA binding	31	31	0.00037	0.034	1.46852108
GO:0003746	F	translation elongation factor activity	31	31	0.00037	0.034	1.46852108
GO:0003690	F	double-stranded DNA binding	40	41	0.00037	0.034	1.46852108
GO:0031982	C	vesicle	64	70	0.002	0.034	1.46852108
GO:0006289	P	nucleotide-excision repair	26	26	0.0013	0.035	1.45593196
GO:0032506	P	cytokinetic process	26	26	0.0013	0.035	1.45593196
GO:0009683	P	indoleacetic acid metabolic process	93	104	0.0014	0.036	1.4436975
GO:0050794	P	regulation of cellular process	3256	4091	0.0014	0.036	1.4436975
GO:0043247	P	telomere maintenance in response to DNA damage	48	51	0.0014	0.037	1.43179828
GO:0006598	P	polyamine catabolic process	34	35	0.0015	0.039	1.40893539
GO:0015996	P	chlorophyll catabolic process	54	58	0.0015	0.039	1.40893539
GO:0046149	P	pigment catabolic process	54	58	0.0015	0.039	1.40893539
GO:0043069	P	negative regulation of programmed cell death	141	162	0.0015	0.039	1.40893539
GO:0009684	P	indoleacetic acid biosynthetic process	92	103	0.0016	0.041	1.38721614
GO:0008170	F	N-methyltransferase activity	39	40	0.00047	0.042	1.37675071
GO:0016903	F	oxidoreductase activity, acting on the aldehyde or oxo group of donors	60	64	0.00047	0.042	1.37675071
GO:0019205	F	nucleobase, nucleoside, nucleotide kinase activity	30	30	0.00048	0.042	1.37675071
GO:0048447	P	sepal morphogenesis	65	71	0.0017	0.043	1.36653154
GO:0048451	P	petal formation	65	71	0.0017	0.043	1.36653154
GO:0048453	P	sepal formation	65	71	0.0017	0.043	1.36653154
GO:0043543	P	protein amino acid acylation	434	524	0.0017	0.044	1.35654732
GO:0006740	P	NADPH regeneration	172	200	0.0017	0.045	1.34678749
GO:0006323	P	DNA packaging	70	77	0.0018	0.047	1.32790214
GO:0009295	C	nucleoid	38	40	0.0029	0.049	1.30980392
GO:0006098	P	pentose-phosphate shunt	171	199	0.0019	0.05	1.30103

Table S15 GO terms for genes in PS3 (Viridiplantae)

GO term	Ontology	Description	Number in input list	Number in BG/Ref	p-value	FDR	-log(FDR)
GO:0003700	F	transcription factor activity	256	1371	2.4E-91	5E-89	88.30103
GO:0030528	F	transcription regulator activity	258	1541	1E-81	1.1E-79	78.9586073
GO:0045449	P	regulation of transcription	220	1139	9.3E-83	1.2E-79	78.9208188
GO:0051171	P	regulation of nitrogen compound metabolic process	223	1184	1.3E-81	8.1E-79	78.091515
GO:0019219	P	regulation of nucleobase, nucleoside, nucleotide and nucleic acid metabolic process	221	1168	2.3E-81	9.9E-79	78.0043648
GO:0010556	P	regulation of macromolecule biosynthetic process	220	1173	2.6E-80	8.5E-78	77.0705811
GO:0009889	P	regulation of biosynthetic process	222	1204	1.6E-79	3.4E-77	76.4685211
GO:0031326	P	regulation of cellular biosynthetic process	222	1204	1.6E-79	3.4E-77	76.4685211
GO:0080090	P	regulation of primary metabolic process	223	1234	3.5E-78	6.5E-76	75.1870866
GO:0006350	P	transcription	221	1222	1.3E-77	2E-75	74.69897
GO:0003677	F	DNA binding	280	1868	5.4E-77	3.7E-75	74.4317983
GO:0010468	P	regulation of gene expression	222	1246	1E-76	1.5E-74	73.8239087
GO:0031323	P	regulation of cellular metabolic process	225	1290	7.3E-76	9.4E-74	73.0268721
GO:0060255	P	regulation of macromolecule metabolic process	223	1284	6.4E-75	7.5E-73	72.1249387
GO:0019222	P	regulation of metabolic process	226	1382	6.5E-71	7E-69	68.154902
GO:0006355	P	regulation of transcription, DNA-dependent	143	598	1.6E-68	1.6E-66	65.79588
GO:0051252	P	regulation of RNA metabolic process	143	603	5.1E-68	4.7E-66	65.3279021
GO:0006351	P	transcription, DNA-dependent	143	630	2E-65	1.7E-63	62.7695511
GO:0032774	P	RNA biosynthetic process	143	631	2.5E-65	2E-63	62.69897
GO:0003676	F	nucleic acid binding	289	2684	1E-49	5.2E-48	47.2839967
GO:0034645	P	cellular macromolecule biosynthetic process	231	1946	5.8E-48	4.4E-46	45.3565473
GO:0009059	P	macromolecule biosynthetic process	231	1967	3.2E-47	2.3E-45	44.6382722
GO:0006139	P	nucleobase, nucleoside, nucleotide and nucleic acid metabolic process	234	2044	5.9E-46	4E-44	43.39794
GO:0010467	P	gene expression	233	2037	9.8E-46	6.3E-44	43.2006595
GO:0050794	P	regulation of cellular process	239	2157	1.7E-44	1E-42	42
GO:0050789	P	regulation of biological process	248	2362	4E-42	2.4E-40	39.6197888
GO:0016070	P	RNA metabolic process	145	994	5.3E-42	3E-40	39.5228787
GO:0065007	P	biological regulation	256	2735	2E-35	1.1E-33	32.9586073
GO:0006807	P	nitrogen compound metabolic process	237	2466	6.5E-35	3.4E-33	32.4685211
GO:0005634	C	nucleus	186	1763	2.1E-33	6.8E-31	30.1674911
GO:0044249	P	cellular biosynthetic process	240	2850	2.1E-27	1E-25	25
GO:0009873	P	ethylene mediated signaling pathway	26	46	1.4E-25	6.7E-24	23.1739252
GO:0071369	P	cellular response to ethylene stimulus	26	46	1.4E-25	6.7E-24	23.1739252
GO:0000160	P	two-component signal transduction system (phosphorelay)	28	58	7.3E-25	3.2E-23	22.49485
GO:0009058	P	biosynthetic process	240	3014	2.4E-24	1E-22	22
GO:0044260	P	cellular macromolecule metabolic process	251	3850	2.8E-15	1.2E-13	12.9208188
GO:0043231	C	intracellular membrane-bounded organelle	334	5480	3.4E-15	3.7E-13	12.4317983
GO:0043227	C	membrane-bounded organelle	335	5488	2.7E-15	3.7E-13	12.4317983
GO:0009723	P	response to ethylene stimulus	28	128	2.6E-14	1E-12	12
GO:0043229	C	intracellular organelle	347	5841	2.7E-14	1.7E-12	11.7695511

GO:0043226	C	organelle	347	5842	2.7E-14	1.7E-12	11.7695511
GO:0016563	F	transcription activator activity	25	116	8.4E-13	3.5E-11	10.455932
GO:0071495	P	cellular response to endogenous stimulus	35	237	4E-12	1.6E-10	9.79588002
GO:0009755	P	hormone-mediated signaling pathway	33	215	5.4E-12	2E-10	9.69897
GO:0032870	P	cellular response to hormone stimulus	33	215	5.4E-12	2E-10	9.69897
GO:0043170	P	macromolecule metabolic process	254	4246	7.7E-12	2.8E-10	9.55284197
GO:0071310	P	cellular response to organic substance	38	297	4.2E-11	1.5E-09	8.82390874
GO:0070887	P	cellular response to chemical stimulus	38	320	3.6E-10	1.2E-08	7.92081875
GO:0023034	P	intracellular signaling pathway	27	179	6.1E-10	2E-08	7.69897
GO:0044424	C	intracellular part	355	6602	5.6E-10	3E-08	7.52287875
GO:0005622	C	intracellular	364	6862	1.4E-09	6.6E-08	7.18045606
GO:0042991	P	transcription factor import into nucleus	6	6	2.3E-09	7.5E-08	7.12493874
GO:0016564	F	transcription repressor activity	13	44	3.7E-09	1.3E-07	6.88605665
GO:0034357	C	photosynthetic membrane	29	220	3.6E-09	1.4E-07	6.85387196
GO:0009579	C	thylakoid	33	299	2.8E-08	0.000001	6
GO:0044436	C	thylakoid part	29	245	0.00000004	0.000001	6
GO:0055035	C	plastid thylakoid membrane	25	190	4.1E-08	0.000001	6
GO:0042651	C	thylakoid membrane	26	203	3.9E-08	0.000001	6
GO:0009535	C	chloroplast thylakoid membrane	25	190	4.1E-08	0.000001	6
GO:0031976	C	plastid thylakoid	28	236	6.4E-08	0.0000014	5.85387196
GO:0009534	C	chloroplast thylakoid	28	236	6.4E-08	0.0000014	5.85387196
GO:0031984	C	organelle subcompartment	28	238	7.7E-08	0.0000015	5.82390874
GO:0010200	P	response to chitin	19	118	6.5E-08	0.000002	5.69897
GO:0023033	P	signaling pathway	27	226	9.4E-08	0.0000029	5.537602
GO:0005488	F	binding	365	7226	0.0000001	0.0000031	5.50863831
GO:0009743	P	response to carbohydrate stimulus	23	184	0.00000035	0.000011	4.95860731
GO:0044237	P	cellular metabolic process	276	5375	0.00000049	0.000014	4.85387196
GO:0007242	P	intracellular signaling cascade	40	463	0.00000083	0.000024	4.61978876
GO:0048825	P	cotyledon development	7	20	0.0000043	0.00012	3.92081875
GO:0009522	C	photosystem I	6	15	0.0000088	0.00017	3.76955108
GO:0044455	C	mitochondrial membrane part	11	61	0.000012	0.00021	3.67778071
GO:0010033	P	response to organic substance	62	946	0.000015	0.00041	3.38721614
GO:0009725	P	response to hormone stimulus	47	677	0.000035	0.00094	3.02687215
GO:0044238	P	primary metabolic process	267	5557	0.000043	0.0011	2.95860731
GO:0009521	C	photosystem	8	39	0.000069	0.0012	2.92081875
GO:0017038	P	protein import	11	71	0.000051	0.0013	2.88605665
GO:0031090	C	organelle membrane	43	624	0.000083	0.0013	2.88605665
GO:0004221	F	ubiquitin thiolesterase activity	8	38	0.000057	0.0015	2.82390874
GO:0033365	P	protein localization in organelle	10	65	0.00012	0.003	2.52287875
GO:0043565	F	sequence-specific DNA binding	10	66	0.00013	0.0031	2.50863831
GO:0009719	P	response to endogenous stimulus	48	741	0.00015	0.0037	2.43179828
GO:0048510	P	regulation of timing of transition from vegetative to reproductive phase	5	16	0.0002	0.0047	2.32790214

GO:0048506 P	regulation of timing of meristematic phase transition	5	16	0.0002	0.0047	2.32790214
GO:0051170 P	nuclear import	7	34	0.00019	0.0047	2.32790214
GO:0009987 P	cellular process	339	7428	0.00022	0.005	2.30103
GO:0009798 P	axis specification	6	25	0.00023	0.0051	2.29242982
GO:0015992 P	proton transport	7	36	0.00028	0.0061	2.21467016
GO:0006818 P	hydrogen transport	7	36	0.00028	0.0061	2.21467016
GO:0051716 P	cellular response to stimulus	40	602	0.00029	0.0063	2.20065945
GO:0044434 C	chloroplast part	39	600	0.00052	0.0079	2.10237291
GO:0051239 P	regulation of multicellular organismal process	17	185	0.00049	0.01	2
GO:0044435 C	plastid part	41	653	0.00075	0.011	1.95860731
GO:0044464 C	cell part	461	10564	0.00079	0.011	1.95860731
GO:0005623 C	cell	461	10564	0.00079	0.011	1.95860731
GO:0031978 C	plastid thylakoid lumen	9	69	0.00089	0.011	1.95860731
GO:0009543 C	chloroplast thylakoid lumen	9	69	0.00089	0.011	1.95860731
GO:0009507 C	chloroplast	105	2109	0.0016	0.019	1.7212464
GO:0031977 C	thylakoid lumen	9	75	0.0016	0.019	1.7212464
GO:0031966 C	mitochondrial membrane	14	156	0.0018	0.02	1.69897
GO:0006606 P	protein import into nucleus	6	33	0.0011	0.023	1.63827216
GO:0048518 P	positive regulation of biological process	16	182	0.0011	0.023	1.63827216
GO:0048827 P	phyllome development	16	184	0.0013	0.026	1.58502665
GO:0005516 F	calmodulin binding	15	167	0.0013	0.027	1.56863624
GO:0015078 F	hydrogen ion transmembrane transporter activity	10	89	0.0015	0.029	1.537602
GO:0010224 P	response to UV-B	6	35	0.0015	0.03	1.52287875
GO:0009536 C	plastid	109	2247	0.0029	0.031	1.50863831
GO:0006605 P	protein targeting	12	121	0.0016	0.032	1.49485002
GO:0034220 P	ion transmembrane transport	6	36	0.0018	0.034	1.46852108
GO:0034504 P	protein localization in nucleus	6	36	0.0018	0.034	1.46852108
GO:0005740 C	mitochondrial envelope	14	166	0.0032	0.034	1.46852108
GO:0070469 C	respiratory chain	8	69	0.0036	0.036	1.4436975
GO:0040034 P	regulation of development, heterochronic	5	26	0.0022	0.041	1.38721614
GO:0009893 P	positive regulation of metabolic process	8	66	0.0027	0.049	1.30980392
GO:0031325 P	positive regulation of cellular metabolic process	8	66	0.0027	0.049	1.30980392

Table S16 GO terms for genes in PS4 (Embryophyta)

GO term	Ontology	Description	Number in input list	Number in BG/Ref	p-value	FDR	-log(FDR)
GO:0009733	P	response to auxin stimulus	108	258	1.3E-32	3.1E-29	28.5086383
GO:0045449	P	regulation of transcription	277	1139	3.6E-28	4.3E-25	24.3665315
GO:0010556	P	regulation of macromolecule biosynthetic process	280	1173	4E-27	3.3E-24	23.4814861
GO:0019219	P	regulation of nucleobase, nucleoside, nucleotide and nucleic acid metabolic process	277	1168	2E-26	1.2E-23	22.9208188
GO:0006350	P	transcription	284	1222	1.4E-25	5.9E-23	22.229148
GO:0051171	P	regulation of nitrogen compound metabolic process	277	1184	1.7E-25	5.9E-23	22.229148
GO:0010468	P	regulation of gene expression	288	1246	1.6E-25	5.9E-23	22.229148
GO:0009889	P	regulation of biosynthetic process	280	1204	2.5E-25	6.8E-23	22.1674911
GO:0031326	P	regulation of cellular biosynthetic process	280	1204	2.5E-25	6.8E-23	22.1674911
GO:0080090	P	regulation of primary metabolic process	282	1234	2.7E-24	6.4E-22	21.19382
GO:0060255	P	regulation of macromolecule metabolic process	289	1284	8.5E-24	1.9E-21	20.7212464
GO:0031323	P	regulation of cellular metabolic process	282	1290	2E-21	4E-19	18.39794
GO:0019222	P	regulation of metabolic process	292	1382	6.4E-20	1.2E-17	16.9208188
GO:0050794	P	regulation of cellular process	399	2157	4.1E-16	7.1E-14	13.1487417
GO:0009719	P	response to endogenous stimulus	167	741	6.5E-15	1.1E-12	11.9586073
GO:0010033	P	response to organic substance	200	946	1.7E-14	2.5E-12	11.60206
GO:0009725	P	response to hormone stimulus	154	677	2.9E-14	4.1E-12	11.3872161
GO:0050789	P	regulation of biological process	415	2362	2.4E-13	3.3E-11	10.4814861
GO:0007275	P	multicellular organismal development	260	1433	1.3E-10	1.7E-08	7.76955108
GO:0032502	P	developmental process	279	1570	2.7E-10	3.3E-08	7.48148606
GO:0032501	P	multicellular organismal process	265	1496	9.3E-10	0.00000011	6.95860731
GO:0065007	P	biological regulation	430	2735	0.0000001	0.000011	4.95860731
GO:0045962	P	positive regulation of development, heterochronic	7	7	0.00000039	0.00004	4.39794001
GO:0007568	P	aging	23	68	0.0000028	0.00028	3.55284197
GO:0048513	P	organ development	111	603	0.0000073	0.0007	3.15490196
GO:0048731	P	system development	111	604	0.0000078	0.00073	3.13667714
GO:0010467	P	gene expression	311	2037	0.000033	0.0029	2.537602
GO:0034645	P	cellular macromolecule biosynthetic process	298	1946	0.000037	0.0032	2.49485002
GO:0002376	P	immune system process	53	250	0.000039	0.0032	2.49485002
GO:0006955	P	immune response	52	249	0.000069	0.0055	2.25963731
GO:0048366	P	leaf development	39	171	0.000074	0.0057	2.24412514
GO:0009059	P	macromolecule biosynthetic process	298	1967	0.000077	0.0058	2.23657201
GO:0009887	P	organ morphogenesis	36	154	0.00008	0.0058	2.23657201
GO:0048827	P	phyllome development	41	184	0.000086	0.0059	2.22914799
GO:0042221	P	response to chemical stimulus	231	1484	0.000085	0.0059	2.22914799
GO:0006139	P	nucleobase, nucleoside, nucleotide and nucleic acid metabolic process	305	2044	0.00018	0.012	1.92081875
GO:0048527	P	lateral root development	15	46	0.00023	0.015	1.82390874
GO:0010014	P	meristem initiation	9	20	0.00027	0.017	1.76955108
GO:0045087	P	innate immune response	47	233	0.00034	0.021	1.67778071
GO:0009965	P	leaf morphogenesis	25	102	0.00043	0.026	1.58502665
GO:0048528	P	post-embryonic root development	15	49	0.00049	0.029	1.537602
GO:0040034	P	regulation of development, heterochronic	10	26	0.00057	0.033	1.48148606
GO:0010016	P	shoot morphogenesis	31	141	0.00073	0.041	1.38721614
GO:0031225	C	anchored to membrane	76	202	3E-20	1.3E-17	16.8860566
GO:0031224	C	intrinsic to membrane	117	586	6.7E-08	0.000014	4.85387196

Table S17 GO terms for genes in PS5 (Tracheophyta)

GO term	Ontology	Description	Number in input list	Number in BG/Ref	p-value	FDR	-log(FDR)
GO:0012505	C	endomembrane system	150	1997	1.50E-12	2.70E-10	9.56863624
GO:0031225	C	anchored to membrane	32	202	3.00E-11	2.70E-09	8.56863624
GO:0055044	C	symplast	7	15	6.90E-07	3.10E-05	4.50863831
GO:0009506	C	plasmodesma	7	15	6.90E-07	3.10E-05	4.50863831
GO:0005911	C	cell-cell junction	7	17	2.00E-06	7.10E-05	4.14874165
GO:0030054	C	cell junction	7	18	3.10E-06	9.30E-05	4.03151705

Table S18 GO terms for genes in PS6 (Magnoliophyta)

GO term	Ontology	Description	Number in input list	Number in BG/Ref	p-value	FDR	-log(FDR)
GO:0046910	F	pectinesterase inhibitor activit	22	49	7.3E-13	1.3E-10	9.88605665
GO:0004857	F	enzyme inhibitor activity	29	121	1.1E-08	0.00000096	6.01772877
GO:0010876	P	lipid localization	28	106	1.8E-09	0.0000014	5.85387196
GO:0006869	P	lipid transport	25	92	6.9E-09	0.0000026	5.58502665
GO:0030599	F	pectinesterase activity	18	102	0.00041	0.025	1.60205999
GO:0008289	F	lipid binding	25	168	0.00055	0.025	1.60205999
GO:0005102	F	receptor binding	6	17	0.00092	0.033	1.48148606

Table S19 GO terms for genes in PS9 (Rosids)

GO term	Ontology	Description	Number in input list	Number in BG/Ref	p-value	FDR	-log(FDR)
GO:0005102	F	receptor binding	12	17	2.8E-18	2E-16	15.69897
GO:0048046	C	apoplast	16	127	5.6E-10	3.5E-08	7.45593196
GO:0005576	C	extracellular region	18	193	6.6E-09	0.00000021	6.67778071
GO:0046910	F	pectinesterase inhibitor activit	10	49	7.5E-09	0.00000027	6.56863624
GO:0004857	F	enzyme inhibitor activity	14	121	1.9E-08	0.00000046	6.33724217
GO:0030599	F	pectinesterase activity	11	102	0.0000012	0.000022	4.65757732
GO:0012505	C	endomembrane system	63	1997	0.0000052	0.00011	3.95860732
GO:0030234	F	enzyme regulator activity	14	261	0.00015	0.0022	2.65757732

Table S20 GO terms for genes in PS10 (Brassicales)

GO term	Ontology	Description	Number in input list	Number in BG/Ref	p-value	FDR	-log(FDR)
GO:0012505	C	endomembrane system	196	1997	5E-21	4.1E-19	18.3872161
GO:0005102	F	receptor binding	12	17	3.9E-13	4.5E-11	10.3467875
GO:0005576	C	extracellular region	26	193	0.00000089	0.000036	4.4436975
GO:0048046	C	apoplast	17	127	0.000068	0.0018	2.74472749
GO:0005199	F	structural constituent of cell w	8	32	0.000071	0.0041	2.38721614
GO:0007267	P	cell-cell signaling	7	25	0.000092	0.039	1.40893539
GO:0004857	F	enzyme inhibitor activity	14	121	0.0012	0.047	1.32790214

Table S21 GO terms for genes in PS11(Arabidopsis)

GO term	Ontology	Description	Number in input list	Number in BG/Ref	p-value	FDR	-log(FDR)
GO:0009341	C	beta-galactosidase complex	7	10	5.5E-13	4.2E-11	10.3767507
GO:0004565	F	beta-galactosidase activity	8	28	1.1E-10	2E-09	8.69897
GO:0015925	F	galactosidase activity	8	32	3.7E-10	3.3E-09	8.48148606

Table S22 GO terms for genes in PS12(Arabidopsis thaliana)

GO term	Ontology	Description	Number in input list	Number in BG/Ref	p-value	FDR	-log(FDR)
GO:0012505	C	endomembrane system	304	1997	6.9E-23	1.2E-20	19.9208188
GO:0005886	C	plasma membrane	220	1302	9E-23	1.2E-20	19.9208188

Table S23 GO terms for genes with dN/dS ratio >1 (compared with *Arabidopsis lyrata*)

GO term	Ontology	Description	Number in input list	Number in BG/Ref	p-value	FDR	-log(FDR)
GO:0010876	P	lipid localization	8	24	4.5E-12	8.9E-10	9.05060999
GO:0012505	C	endomembrane system	43	3416	6.4E-08	0.0000032	5.49485002
GO:0006869	P	lipid transport	8	163	0.0000035	0.00035	3.45593196
GO:0008289	F	lipid binding	8	323	0.00037	0.019	1.7212464

Table S24 The phylostrata of genes with dN/dS>1 compared with *A.lyrata*

Phylostrata	Genes with dN/dS>1	Total genes	Enrichment	P-value
Arabidopsis thaliana	7	1752	0.509416936	0.03151279
Arabidopsis	16	188	10.92001092	2.04652E-12
Brassicales	50	970	6.576178451	5.49984E-27
Rosids	13	352	4.723493932	4.57227E-06
Core eudicotyledons	5	276	2.316745436	0.06678295
Eudicotyledons	8	282	3.607503608	0.001790592
Magnoliophyta	19	1571	1.542758777	0.04016194
Tracheophyta	6	846	0.904568069	0.5039034
Embryophyta	17	2635	0.823188743	0.235058
Viridiplantae	2	782	0.325839036	0.05316776
Eukaryota	40	6503	0.78484927	0.04113868
Cellular organisms	15	9103	0.21020415	7.61169E-21
